# Clinical Applications and Anticancer Effects of Antimicrobial Peptides: From Bench to Bedside

**DOI:** 10.3389/fonc.2022.819563

**Published:** 2022-02-23

**Authors:** Ameneh Jafari, Amirhesam Babajani, Ramin Sarrami Forooshani, Mohsen Yazdani, Mostafa Rezaei-Tavirani

**Affiliations:** ^1^ Student Research Committee, Shahid Beheshti University of Medical Sciences, Tehran, Iran; ^2^ Advanced Therapy Medicinal Product (ATMP) Department, Breast Cancer Research Center, Motamed Cancer Institute, Academic Center for Education, Culture and Research (ACECR), Tehran, Iran; ^3^ School of Medicine, Shahid Beheshti University of Medical Sciences, Tehran, Iran; ^4^ Laboratory of Bioinformatics and Drug Design, Institute of Biochemistry and Biophysics, University of Tehran, Tehran, Iran; ^5^ Proteomics Research Center, Faculty of Paramedical Sciences, Shahid Beheshti University of Medical Sciences, Tehran, Iran

**Keywords:** cancer, antimicrobial peptides (AMPs), anticancer peptides (ACPs), apoptosis, angiogenesis, exosome, mechanism

## Abstract

Cancer is a multifaceted global health issue and one of the leading causes of death worldwide. In recent years, medical science has achieved great advances in the diagnosis and treatment of cancer. Despite the numerous advantages of conventional cancer therapies, there are major drawbacks including severe side effects, toxicities, and drug resistance. Therefore, the urgency of developing new drugs with low cytotoxicity and treatment resistance is increasing. Antimicrobial peptides (AMPs) have attracted attention as a novel therapeutic strategy for the treatment of various cancers, targeting tumor cells with less toxicity to normal tissues. In this review, we present the structure, biological function, and underlying mechanisms of AMPs. The recent experimental studies and clinical trials on anticancer peptides in different cancer types as well as the challenges of their clinical application have also been discussed.

## Introduction

Cancer treatment is still one of the biggest challenges in the public health system globally, with a high mortality rate (1, 2). Current therapeutic strategies, including surgery, radiotherapy, chemotherapy, or a combination of these treatments, will prolong a patient’s life expectancy (3, 4). However, several obstacles can affect or limit their effectiveness. For example, drug access is restricted to the whole tumor volume due to the complexity and heterogeneity within the tumor or the surrounding microenvironment that leads to chemotherapy resistance (1). Another unpleasant problem is the lack of specificity of some anticancer drugs, which causes toxic side effects on healthy cells (5, 6). With the advent of molecular biology, cancer treatment today has shifted from chemotherapy and radiotherapy to molecular targeting of cancer, which prevents damage to healthy tissue on the one hand and more effective treatment of cancer on the other (7, 8). In the quest for new anticancer strategies, some of the most attractive compounds that have been tested in the laboratory and are expected to go beyond the shortcomings of traditional medicines are antimicrobial peptides (AMPs) (8).

**Figure 1:**
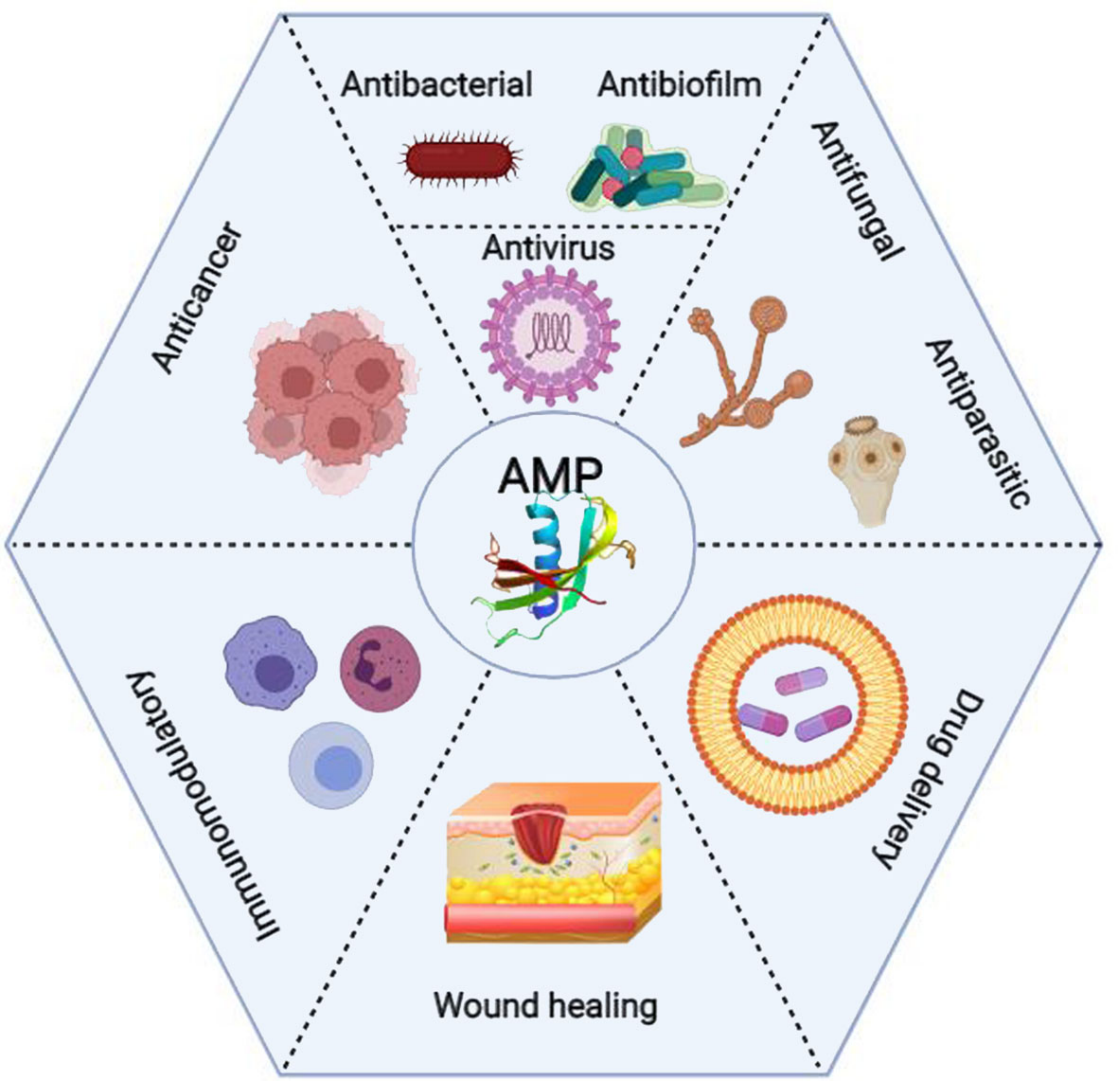
Clinical applications of the antimicrobial peptides (AMPs) from wound healing and drug delivery to anticancer activity (Created with BioRender.com).

These peptides are essential components of the host’s innate immune system and have been found in almost all species of bacteria, fungi, invertebrates, vertebrates, and plants. Virtually any organism secretes AMPs to respond to various pathogens and stress conditions (9, 10). The discovery of AMPs dates back to 1922 when Alexander Fleming discovered the antimicrobial activity of human lysozyme in saliva. More than 5,000 AMPs have been identified so far, either *de novo* or synthesized in the lab (11). AMPs consist of varying lengths (up to 100) of amino acid residues, and they possess broad-spectrum antimicrobial activities against bacteria, viruses, fungi, and protozoa (8, 9). Antibiofilm, anti-inflammatory, and immunomodulatory activities of AMPs have also been reported (12). In addition, AMPs have wound healing properties and can be used in tissue engineering and regenerative medicine. Although most clinical studies focus on the antimicrobial properties of AMPs, many recent pieces of research suggest that they also have anticancer activity (**Figure 1**) (8).

The cytotoxic effects of numerous insect AMPs on different cancerous cell lines, such as breast cancer, lung cancer, melanoma, leukemia, and lymphoma, have been reported (13). These cationic low-molecular-weight AMPs that are involved in both antimicrobial and anticancer activities are termed anticancer peptides (ACPs) (14).

AMPs/ACPs share some common characteristics, including cationicity (positive net charge), high hydrophobicity, and amphipathic structure, giving them an increased affinity for cell membranes (8). Given the very similar characteristics of AMPs/ACPs, efforts have been made to understand why some AMPs have antitumor activity, allowing the better design of ACPs. Due to their features, ACPs could be considered a valuable resource, with a low proclivity to develop cancer cell resistance. The outer membranes of cancer cells have more negative charge molecules than normal cell membranes (15). This feature facilitates the attachment of the ACPs to cancer cells by electrostatic interactions, leading to selective disruption of cancer cell membranes with inducing either necrosis or apoptosis (15). However, ACPs have unique characteristics, including biocompatibility, high therapeutic potency, low risk of emergence in target cells, ease to synthesize and modify, and low toxicity against normal mammalian cells (6, 16–18). Also, these compounds are immunogenic with a short half-life *in vivo* that makes them suitable for clinical applications (13). Considering the molecular characteristics and observed properties, ACPs could be identified or designed as a promising alternative to conventional chemotherapy (19). This review presents the classification, source, structure, biological function, and underlying mechanisms of AMPs/ACPs. Furthermore, we have shed light on the recent experimental studies and clinical trials on ACPs in different cancer types, the challenges and strategies of clinical applications of AMPs/ACPs, and the role of computational approaches in their design.

## The Classifications, Structures, and Characteristics of AMPs

**Figure 2:**
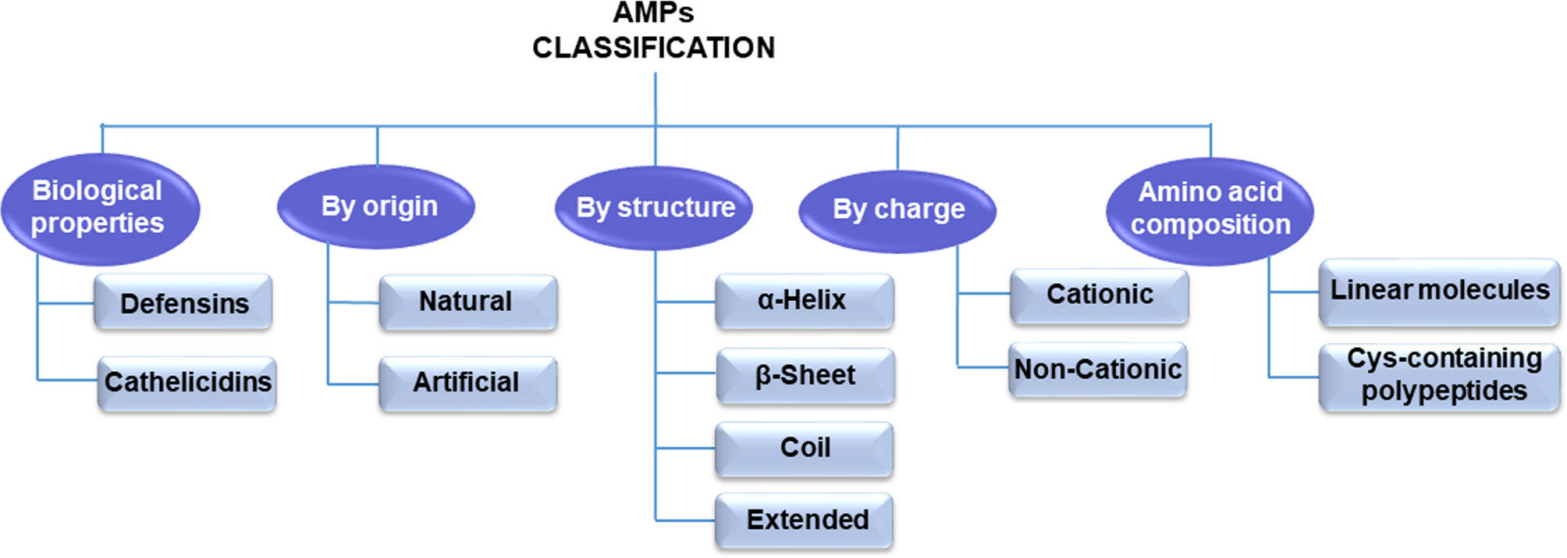
Antimicrobial peptide classification.

Until now, thousands of AMPs have been discovered (20). These peptides are small molecular weight oligopeptides, variable in amino acid composition and host origin. However, they are ubiquitous in nature and are expressed by specific genes. According to the AMP database (http://aps.unmc.edu/AP), there are 3,283 AMPs, of which approximately 259 peptides are listed as anticancer peptides (21). AMPs can be classified into different categories based on these peptides’ various amino acid components, origin, structures, and biological roles (**Figure 2**).

According to the amino acid composition, AMPs are divided into two major types: linear molecules with an α-helical structure without cysteine (e.g., cecropin, magainin) and cysteine-containing polypeptides with disulfide bridge(s) such as insect defensin (22). In another classification, mammals’ AMPs are categorized into cathelicidins and defensins according to their structure and biological properties (23, 24). Natural synthetic or ribosomal synthetic peptides and nonribosomal peptides are other categories for antibacterial peptides (25). Moreover, electrostatic charge is a significant feature for the AMP classification. Therefore, they are classified into two groups based on this feature: cationic peptides and noncationic peptides (26, 27). Since the type, number, and composition of amino acids of AMPs/ACPs play a critical role in their activity, structural classification is currently the most common classification method (28).

AMPs/ACPs can be classified into four categories based on their secondary structures, including α-helical rod conformations, β-sheeted peptides, random coil, and extended structures (29). The peptide chain is generally short and straightforward in the α-helical ACPs (the most common type of ACPs) found widely in the amphibian epidermis (30). They are the most extensively studied type of ACPs at present. The majority of β-pleated sheet ACPs have two or more disulfide bonds and are relatively stable. These ACPs are more complex than α-helical ACPs, and they are often present in plants and animals (31). In random coil ACPs, proline and glycine residues are generally abundant, lacking a typical secondary structure (32). Cyclic ACPs are closed peptides, are more stable than linear structures, and consist of a head-to-tail cyclization backbone or S-S bonds that build cysteine knots (32, 33). Examples of ACPs with different structures are summarized in **Table 1**.

**Table 1:** Some anticancer peptides with different structures and sources.

ACPs name	Structure	Source	Cancer type/cell line	Dosage	Refs
Magainin 2 (MG2)	α-helical	African clawed frog	Bladder cancer/RT4, Breast cancer/MDA-MB-231	198.1 *μ*M, 120 *μ*M	(34, 35)
Aurein	α-helical	Glandular secretions of green and golden bell frogs and southern bell frogs	Glioblastoma/T98G	10^−5^−10^−4^ M	(14, 36)
Buforin IIb	α-helical	Stomach tissue of the Asian toad *Bufo bufo garagrizans*	Leukemia, breast, nonsmall cell lung cancer, prostate, and colon cancer	7.2–23.9 *μ*g/ml	(37)
L-K6	α-helical		Breast cancer/MCF-7	23 μM	(38)
LL37 and FK-16	α-helical	Neutrophils	Colorectal cancer/LoVo and HCT116	∼40 µM	(39)
Brevenin-2R	α-helical	Skin of the frog *Rana ridibunda*	Breast cancer/MCF-7, T-cell leukemia/Jurkat, B-cell lymphoma/BJAB	10–15 μg/ml, 20–25 μg/ml, 30–40 μg/ml	(40)
Polybia-MPI	α-helical	Venom of the social wasp *Polybia paulista*	Prostate cancer/PC-3, bladder cancer/Biu87, and EJ	64.68 μM, 52.16 μM, 75.51 μM	(41)
Dermaseptin B2	α-helical	*Phyllomedusa* frog	Prostate cancer/PC3, DU145 and LnCap	0.71-2.65 μM	(42)
Bovine lactoferricin (LfcinB)	β-pleated sheet	Bovine milk	Stomach cancer cell/SGC-7901	∼100 μ*M*	(43)
MPLfcinB6	β-pleated sheet	Designed	T-leukemia cells/Jurkat and CEM	∼25 μM	(44)
LfcinB-P13	β-pleated sheet	Synthesized	Liver cancer/SMMC772, L02	41.8 µg/ml, >100 µg/ml	(45)
Human neutrophil peptide (HNP-1)	β-pleated sheet		Prostate cancer/PC-3	2.2 μM	(6)
Alloferon	Random coil	Insects	Herpes simplex virus, human papillomavirus	NR	(46)
KW-WK	Random coil	Designed	Human kidney/293 cells	64 µM-128 µM	(33)
PR-35	Random coil	Designed	293T cells	NR	(47)
Diffusa Cytide 1	Cyclic	Leaves and roots of *H. diffusa*	Prostate cancer/LNcap, PC3, DU145	5.03 µM, 2.24 µM, 3.32 µM	(48)
H-10	Cyclic	Mouse malignant melanoma	Melanoma/B16	>10 µM	(49)

NR, Not reported.

## The Biological Function of Antimicrobial Peptides

AMPs have a wide range of activities against various species, including viruses, bacteria, fungi, and even mammalian cells; however, the molecular mechanisms by which they act are frequently not well known. In the literature, AMPs are often referred to as a “promising alternative to antibiotics” that they show a wider range of antibacterial effects than traditional antibiotics (50). In addition to bacteria, they are also effective against pathogens, fungi, and viruses (51). For instance, the human cathelicidin peptide LL37 is cationic and α-helical that possesses antimicrobial activity for bacteria, fungi, mold, protozoa, and some enveloped viruses. The inhibitory effects of AMPs on various DNA and RNA viruses such as influenza virus, HIV, hepatitis B virus, and herpes virus have been demonstrated (52). Moreover, different cationic AMPs can be applied in combination with traditional antibiotics to boost the medicinal effects of each and even expand the antibacterial scope of the latter (49, 51).

**Figure 3:**
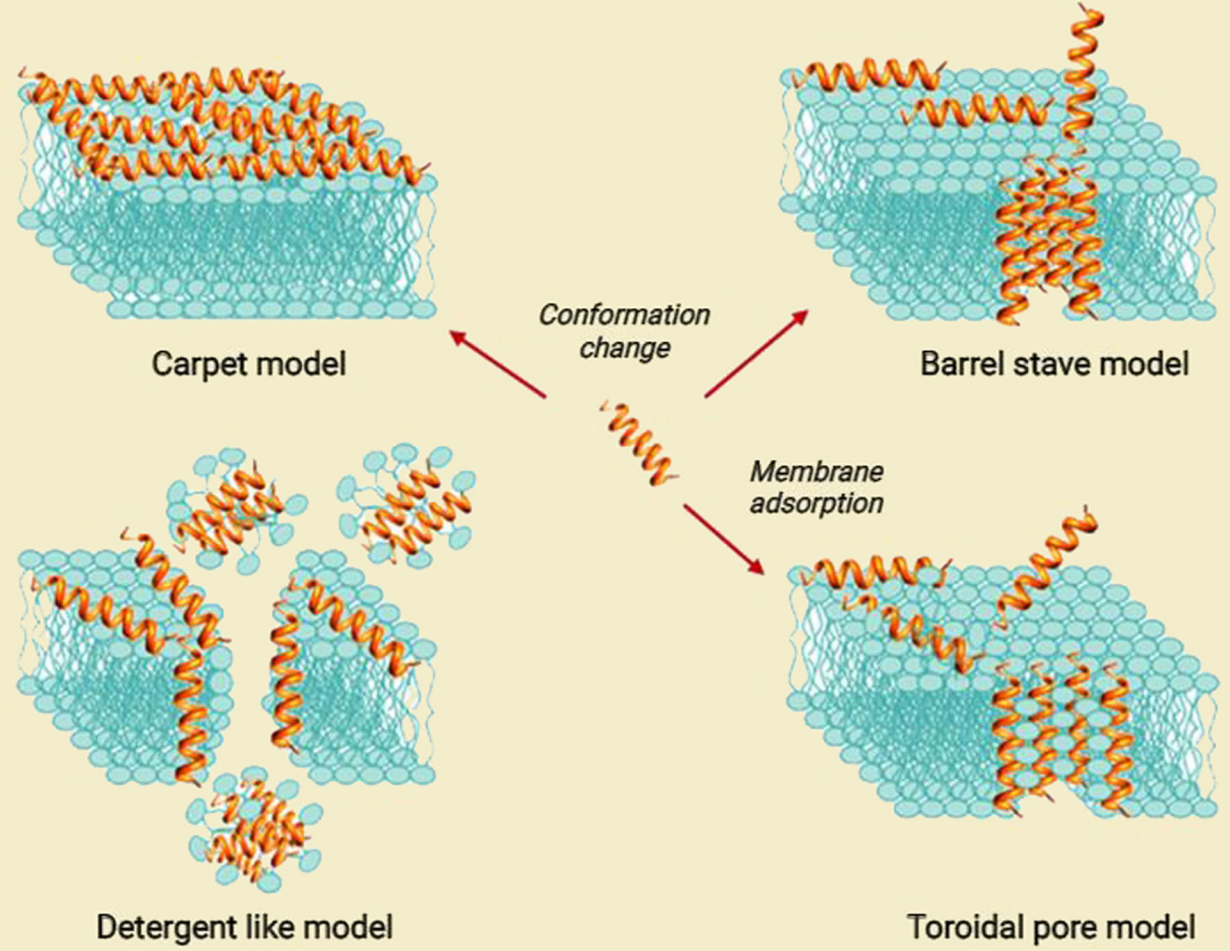
Proposed models for the mode of action for extracellular AMP activity [modified from ref (57)].

AMPs/ACPs play their biological function in different manners. One is the ability of AMPs/ACPs to bind directly with bacterial membranes or cancer cell walls due to their cationic and amphipathic nature (53). Generally, these cationic AMPs/ACPs contain positively charged amino acids like lysine and arginine and possess a net positive charge ranging from +2 to +9 at neutral pH (54, 55). Since normal eukaryotic cell membranes are made of uncharged neutral phospholipids, sphingomyelins, and cholesterol, AMPs/ACPs can exert antimicrobial activity without harming normal cells. AMPs bind to bacteria membranes in different models, including a barrel-stave, carpet-like, toroidal pore model and a detergent-like model (56) (**Figure 3**).

The interaction of AMPs with microbial membranes leads to the killing of microbes through non-enzymatic membrane disruption. Defensins and cathelicidins, the major families of antimicrobial peptides, can destroy the integrity of the membrane by forming pores in the bacterial membrane and then peptide insertion, causing lysis of the targeted microbes and leading to cell death (23, 58). Some AMPs like phospholipase A2 (PLA2) disrupt bacterial membranes through enzymatic digestion. Nevertheless, some peptides kill bacteria by disturbing their normal physiological activity and inhibiting intracellular functions, such as blocking enzyme activity, DNA replication, RNA transcription, and suppressing protein synthesis without damaging the cell membrane (50, 59). Some AMPs can also prevent biofilm formation and disrupt current biofilms (60).

## The Anticancer Mechanisms of ACPs

Despite considerable achievements in treating tumors, there are significant obstacles in controlling the tumor progression and fatal consequences regarding special genetic features of cancerous cells and the tumor microenvironment (TME) condition. Mainly carcinogenesis initiates from alteration in genetic and/or epigenetic cellular characteristics triggering some cellular pathways that induce malignant features in affected cells such as immune evasion, uncontrolled cell division, abnormal metabolism, immortality, and alteration of the cellular structure (61). Considering the fact that plasma membrane dresses the entire cell content and is a representative component to external factors, alteration in plasma membrane components is critical in malignant processes. The plasma membrane structure obeys the fluid-mosaic pattern in which proteins are flowing within a bilayer of phospholipids. In this model, proteins can freely rotate, move laterally, and ascend or descend between plasma layers (62). Although both normal and malignant cells obey the fluid-mosaic pattern in their plasma membrane, there are considerable differences in the membrane composition between normal and malignant cells. In fact, altered TME, such as elevated reactive oxygen species (ROS) and hypoxia, dysregulates phospholipid transporters, altering the regular pattern of plasma membrane phospholipids (63). Particularly, anionic phospholipids such as phosphatidylserine (PS) and phosphatidylethanolamine (PE) move from the inner side to the outer side, resulting in extra membrane negative charge and increased transmembrane potential (64). The membrane surface changes are not limited to enhanced negative charge. It has been shown that the membranes of malignant cells contain a more significant number of microvilli that increase the anchoring area for peptide and other external molecule adhesion (65).

Besides the cellular changes, tumor progression changes the elements of TME. It has been shown that high and rapid consumption of nutrients and oxygen along with the accumulation of metabolic substances confront tumor development; thus, cancer cells adopt compatible features to overcome undesired TME conditions (66). Cancer cells reprogram their metabolic pathways through altering glycolysis-related proteins (GLUT1, GLUT3, LDHA, and PKM2), in which tumor cells prefer fermentation to aerobic, the so-called “Warburg effect” (67, 68). Additionally, oxygen tension affects the expression pattern of slug, N-cadherin, snail, E-cadherin, and vimentin and elevates the amounts of matrix metalloproteinases (MMPs) in TME that finally enhances epithelial–mesenchymal transition (EMT) as an essential factor in tumor metastasis (69, 70). Hypoxia and other TME factors also increase angiogenesis by enhancing the expression of angiogenin, vascular endothelial growth factor (VEGF), TGF‐β, basic fibroblast growth factor (bFGF), focal adhesion kinase (FAK), Src, MMP-2, MMP-9, and platelet-derived growth factor (PDGF) (68, 71). Furthermore, TME contains several infiltrated or resident inflammatory cells and mediators that participate in all neoplasm progression stages, from tumor initiation to cancer promotion and invasion to the near or metastasis to distant tissues (72).

As another problem, chemotherapeutic drug resistance remained a primary limiting factor to achieving desired therapeutic outcomes in patients with cancer. Tumor heterogeneity, immune evasion, condition of TME, expression of efflux pumps, and presence of cancer stem cells are attributed to drug resistance of cancer cells (73, 74). These drugs also affect several body organs that produce undesirable side effects such as fatigue, diarrhea, constipation, chest pain, mucositis, pain, rash, vomiting, and anemia (75). Considering the challenges in cancer therapy, proposing an appropriate and targeted treatment strategy may improve outcomes and increase the life quality.

**Figure 4:**
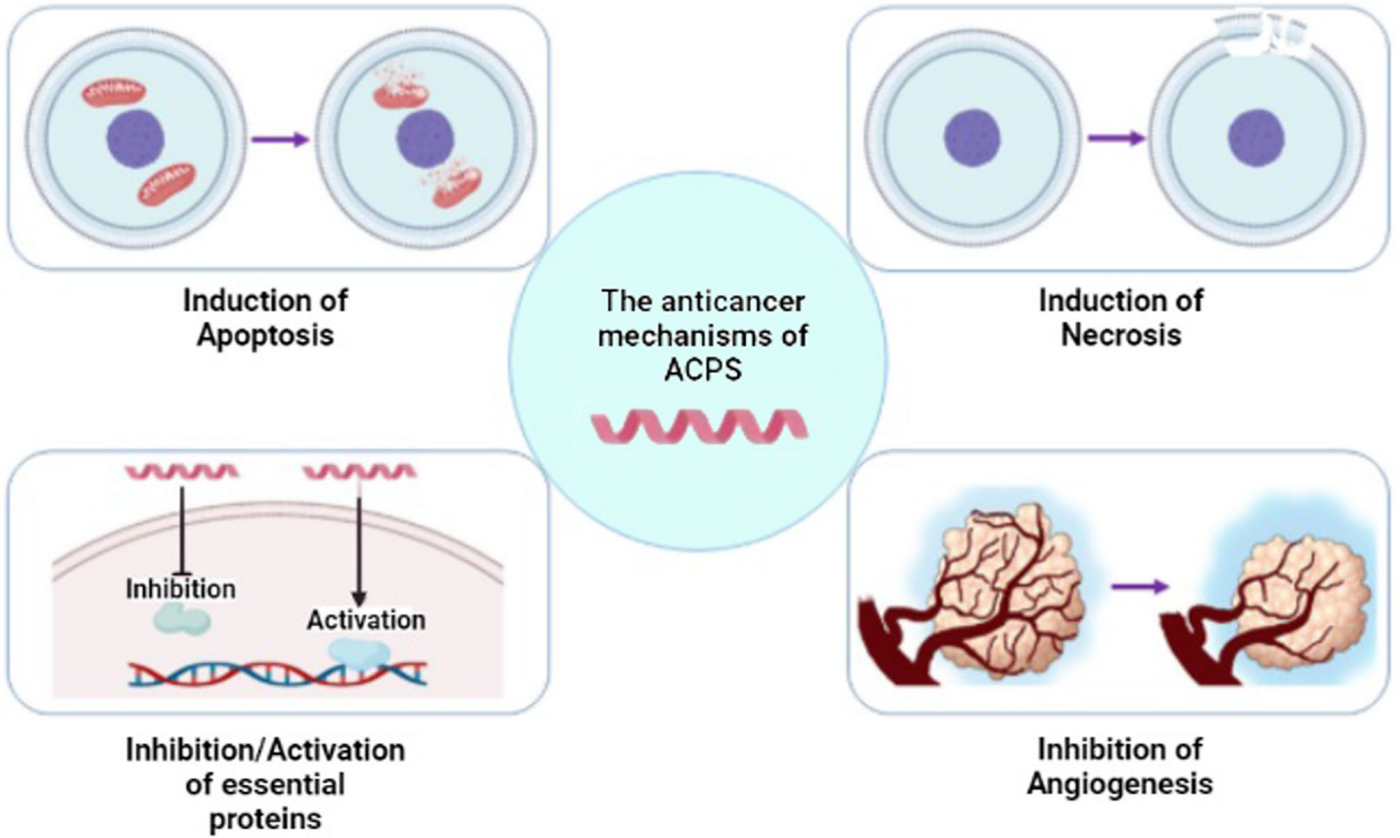
Different mechanisms of anticancer peptide (ACP) function. ACPs can function through a variety of mechanisms, including apoptosis or necrosis induction of cancer cells, activation/inhibition of proteins, and inhibition of angiogenesis [modified from ref (87)]. (Created with BioRender.com).

Recently, studies suggest using AMPs as a novel therapeutic approach for cancer treatment. Many studies reported that AMPs selectively induce apoptosis and necrosis of cancer cells by causing membrane lysis or pore formation (15, 76, 77). Negative outer membrane charge due to anionic phospholipids increases the interaction of cationic AMP and anionic cancer cells (78, 79). In addition to the anionic phospholipids of the cancer cell membrane, the concentration of some anionic glycoproteins such as mucins and heparan sulfate proteoglycans increases in the neoplastic situation, which results in the enhancement of AMP–cancer cell membrane interaction (16, 80, 81). Besides, the presence of more microvilli compared to normal cells facilitates the interaction of AMPs and cell membrane (15). The next step following AMP–membrane interaction is AMP penetration into the targeted cancer cell. AMPs enter the cells in two distinctive manners: energy-dependent or energy-independent. It has been suggested that AMPs containing amino acids with a positive charge, including arginine and lysine, select an energy-dependent way, while the other AMPs, including MMGP1 and maganin, reach inside the cancer cell *via* energy-independent direct cell penetration (82–84). Following the attachment of AMPs to the cancer cell membrane, they can induce antineoplastic effects *via* altering the integrity of the cancer cell membrane, changing some intracellular pathways, inhibiting angiogenesis pathway, and affecting the immune system, which depends on the AMP and cancer types (85, 86). Various mechanisms of action of ACPs is shown in **Figure 4**. Considering the heterogeneous features of cancer cells in different malignancies, the effectiveness of AMPs, action mechanisms, and possible use in cancer therapy in various cancer types are discussed in the next section.

## The Antineoplastic Roles of AMPs in Various Cancer Types

### Urinary Bladder Cancer

Urinary bladder cancer (UBC) is a heterogeneous disease faced with undesirable clinical outcomes due to insufficient research, poor understanding of cancer biology, and lack of novel therapeutic approaches (88). Almost 3.0% of all newly diagnosed cancer cases and 2.1% of all cancer mortality are attributable to UBC (89). Depending on the stage, the main chemotherapeutic regimes consist of methotrexate, vinblastine, and cisplatin in addition to anthracycline such as doxorubicin or epirubicin (90). However, the therapeutic results and adverse effects are unsatisfactory.

Several AMPs have shown therapeutic effects against UBC by interfering with intracellular pathways or disrupting cell membranes. It has been demonstrated that Cecropin A and Cecropin B can play an antineoplastic role by reducing tumor cell proliferation, interrupting DNA synthesis, and lysing tumor cells directly (91). One study indicated that Magainin II could repress bladder cancer cell proliferation in a dose-dependent manner unaffected by the multidrug resistance (MDR) phenotype. Magainin II induced cytotoxic effects against all bladder cancer cell lines by an average IC50 of 198.1 μM (range, 52.4–484.03 μM) based on the WST-1 assay and 75.2 μM (range, 31.0–135.3 μM) based on the BrdU assay (34). Wang et al. demonstrated that polybia-mastoparan I (MPI) derived from the venom of the social wasp Polybia Paulista prevented the proliferation of bladder and prostate tumor cells *via* cell membrane disrupting. The cytotoxic effect was limited to the neoplastic cells, and the proteins showed cell selectivity action (92).

In order to increase the efficacy, studies tried to optimize the antineoplastic effects of AMPs and develop new therapeutic application methods. It is indicated that polybia encounters difficulties in transmucosal penetration when administered in an intravesical way. The low penetration rate is attributed to the bladder wall’s particular histology and the high molecular weight and hydrophilicity of polybia-MPI that could limit the therapeutic effect. Li et al. synthesized fluorinated polyethylenimine (F-PEI) to design an effective formulation for polybia-MPI, which displayed significant cross-membrane, transmucosal, and intratumoral penetration ability due to hydrophobic and lipophobic features of fluorinated chains in F-PEI. Intravesical administration of polybia-MPI/F-PEI nanoparticles in mice with orthotopic bladder tumors resulted in a prolonged lifetime within four weeks (survival rate of 83% for the MPI/F-PEI group compared to 33% for control groups) and repressed tumor growth. In this regard, the tumor volume ratio after and before the treatment (Vt : Vo) in the control group was 13, while it was reduced to 2 in the treatment group (93). In another study, Huang et al. designed a cancer vaccine using pardaxin (GE33) as an effective antineoplastic peptide. They showed that *in vivo* administration of pardaxin combined with inactivated mouse bladder tumor cell lysates improves nitrous oxide (NO) secretion (94). It has been indicated that NO can induce antitumorigenic effects depending on the source, the level of production, and the tumor microenvironment (95). Besides, immunized mice showed prevented growth of the tumor and improved recruitment of cytotoxic T cells, monocytes, T-helper cells, and NK cells. It seemed that increased levels of monocyte chemoattractant protein-1 (MCP-1), IL-6, and IL-12 in mouse macrophages facilitated the increased immune response. In addition to the influential inflammatory role of pardaxin, decreased VEGF expression was also observed (94).

Collectively, promising preclinical results of using AMPs in UBC encourage scientists to translate AMPs into clinical practice. However, some critical questions on the safety and efficacy of AMPs in UBC treatment and appropriate dosage remained unsolved that should be evaluated before introducing AMPs to clinical practice. Although modification of AMPs to increase stability, efficacy, and bioavailability was assessed in some studies, there is an essential requirement for developing new approaches in AMP modification.

### Breast Cancer

Based on the National Cancer Institute, breast cancer is the second main reason for women’s death in the USA. Different classification of breast cancer is used to specify therapeutically and follow up approaches. Based on the stage of disease and characteristics of the tumor, many chemotherapeutic drugs such as cyclophosphamide, doxorubicin, taxanes, as well as hormone-based and monoclonal antibody drugs have been administered to treat breast cancer (96, 97). However, the heterogeneous nature of breast cancer and impressive side effects of therapeutic interventions have reduced the success rate of the treatments.

AMPs induce antineoplastic features on breast cancer cells through several mechanisms, including cell membrane disruption, mitochondrial-dependent apoptosis, triggering some intracellular pathways, and disrupting the nucleus. It has been shown that mammal-derived AMPs can display cytotoxic effects against breast cancer cells. For instance, peptides derived from bovine lactoferricin (LfcinB), a peptide fragment of bovine lactoferrin, were cytotoxic for breast cancer cells in a concentration-dependent manner that was optimum in a concentration of 22 µM (98). In a bioinformatics study, E-kobon et al. evaluated the cytotoxicity of six HPLC-separated fractions of the giant African snails, *Achatina fulica*, mucus against the MCF-7 breast cancer cells. Their HPLC evaluation showed that the mucous of giant African snails contains six peaks (fraction) named F1, F2, F3, F4, F5, and F6. They found that 16 proteins in F2 and F5 fractions induced significant cytotoxicity on MCF-7 cell lines. They showed that these small cationic amphipathic peptides could be hopeful agents for novel anticancer drug development (99). Hsiao et al. showed that peptide derivatives of Ixosin-B-amide, isolated from salivary glands of the hard tick I, can induce cytotoxic effects on breast cancer cells. They demonstrated that MAP-04-03 had antiproliferative effects on breast cancer cells. Additionally, it inhibited cancer cell migration at lower concentrations (100, 101).

Previously, pore formation was considered the main underlying reason for cell death, high membrane permeabilization, or bilayer disruption, while recent studies suggest new mechanisms (64, 102). It has been shown that defensin, derived from avocado, could alter the gene expression pattern of the intrinsic apoptosis pathway factors such as Apaf-1, cytochrome c, and caspase 7 and 9 (103). Besides, defensin persuaded the loss of the inner mitochondrial transmembrane potential and increased the phosphorylation of MAPK p38, which resulted in cancer cell death (103). Another study proved the role of intracellular cytotoxic mechanisms of AMPs. The authors reported that tilapia piscidin 4 (TP4), derived from *Oreochromis niloticus*, induces an activator protein-1 (AP-1) protein named FosB through disrupting calcium homeostasis in triple-negative breast cancer (TNBC) cells (104). FosB, a Fos family member, can dimerize with proteins of the JUN family and consequently forms AP-1 (104). Increased levels of Fos and JUN proteins in cooperation with AP-1 have been stated in situations in which cells undergo apoptosis (105, 106). Therefore, FosB overexpression can increase TNBC cell apoptosis (104, 107). Wang et al. reported that L-K6 as an AMP only amplified membrane permeability while not disrupting the cancer cell membrane. Proving the intact consistency of the membrane, they suggested that L-K6 cytotoxicity might be attributed to the intracellular biofunctions of this AMP. In this line, minor mitochondrial membrane depolarization and no cytoskeleton disruption were observed that exclude these reasons. However, it was indicated that L-K6 translocated into the cell nucleus and accordingly disrupted the cancer cell nucleus (33).

On the other hand, some AMPs affect cancer cells in caspase-independent and -dependent manner. Wang and colleagues showed that temporin-1CEa could induce cancer cell death in caspase-independent and -dependent pathways. Temporin-1CEa disrupts the cancer cell membrane, resulting in cell-surface phosphatidylserine exposure, a rise of plasma membrane permeability, and quick transmembrane potential depolarization. This AMP also worked through mitochondrial-dependent pathways, including uncontrolled intracellular calcium leakage, alteration of mitochondrial membrane potential, and overproduction of ROS. It seemed that higher concentrations of AMPs induce cell membrane disruption, while lower concentrations induce cell death *via* mitochondrial-dependent pathways (108).

Administrating AMPs combined with chemotherapeutic drugs can be used as a new therapeutic approach. In this way, some studies have suggested that AMPs may show chemosensitizing properties. It has been concluded that sublethal doses of NRC-03 and NRC-07 meaningfully reduced the half-maximal effective concentration (EC50) of cisplatin for breast carcinoma cells. Pretreatment of NRC-03 and NRC-0 diminished the EC50 of cisplatin by 5.5- and 1.6-folds, respectively. It seems that these AMPs destroy the integrity of the nuclear membrane that provides facilitated access of cisplatin and other DNA-related chemotherapeutic agents to the cancer cell nucleus (109). In another study, administrating doxorubicin (dose: 6 μg/ml) combined with nisin (dose: 10 μg/ml) at sub-inhibitory concentrations resulted in threefold higher cytotoxicity than drug or AMP alone. Increased doxorubicin uptake by breast cancer cells has also been concluded due to the instability of cell membranes or poration (110).

Controlling considerable complications of breast cancer needs introducing new therapeutic approaches. Considering the fact that surgeries, especially total mastectomy, significantly impress the life quality of patients, taking nonsurgical interventions can be a desirable approach (111). AMPs have shown potent antineoplastic effects in breast cancers in both mitochondrial-dependent and mitochondrial-independent manners. Besides, the combination therapy of AMPs with chemotherapeutic drugs was studied by several scientists (109, 110). Thus, using AMPs combined with conventional therapeutic approaches such as radiotherapy and chemotherapy may increase the hope for better patient outcomes. However, further studies are required for determining possible side effects and appropriate dosage and route of delivery.

### Colorectal Cancer

Colorectal cancer is the second leading cause of cancer mortality in the United States (112). Colorectal carcinomas are classified as familial, sporadic, and inherited based on different mutations in specific genes such as oncogenes, tumor suppressor genes, and DNA repair genes. The broad spectrum of involved gene mutations and molecular pathways, as well as the heterogeneity of colorectal cancer, makes its treatment challenging (113).

Antineoplastic effects of AMPs against colorectal cancer cells have been widely studied. Different mechanisms are suggested for AMP actions in colorectal cancer, including inducing cytotoxicity, changing metabolic profile, cell cycle regulatory proteins, and activity of microRNAs. It has been shown that the BmKn2 peptide from scorpion has a cytotoxic effect against human colon cancer cells (114). In another study, intratumor administrating of 5 ng microcin E492 (MccE492), a bacteriocin released from *Klebsiella pneumonia*, reduced the xenograft colorectal tumor cell mass in the zebrafish model 3.5 times more compared to the control group (115). Nisin, a potent bacteriocin and antibacterial peptide, attenuated the proliferation of colon adenocarcinoma cells and suppressed metastatic progression by downregulating carcinoembryonic antigen (CEA), carcinoembryonic cell adhesion molecule 6 (CEAM6), MMP2F, and MMP9F genes (116).

**Figure 5:**
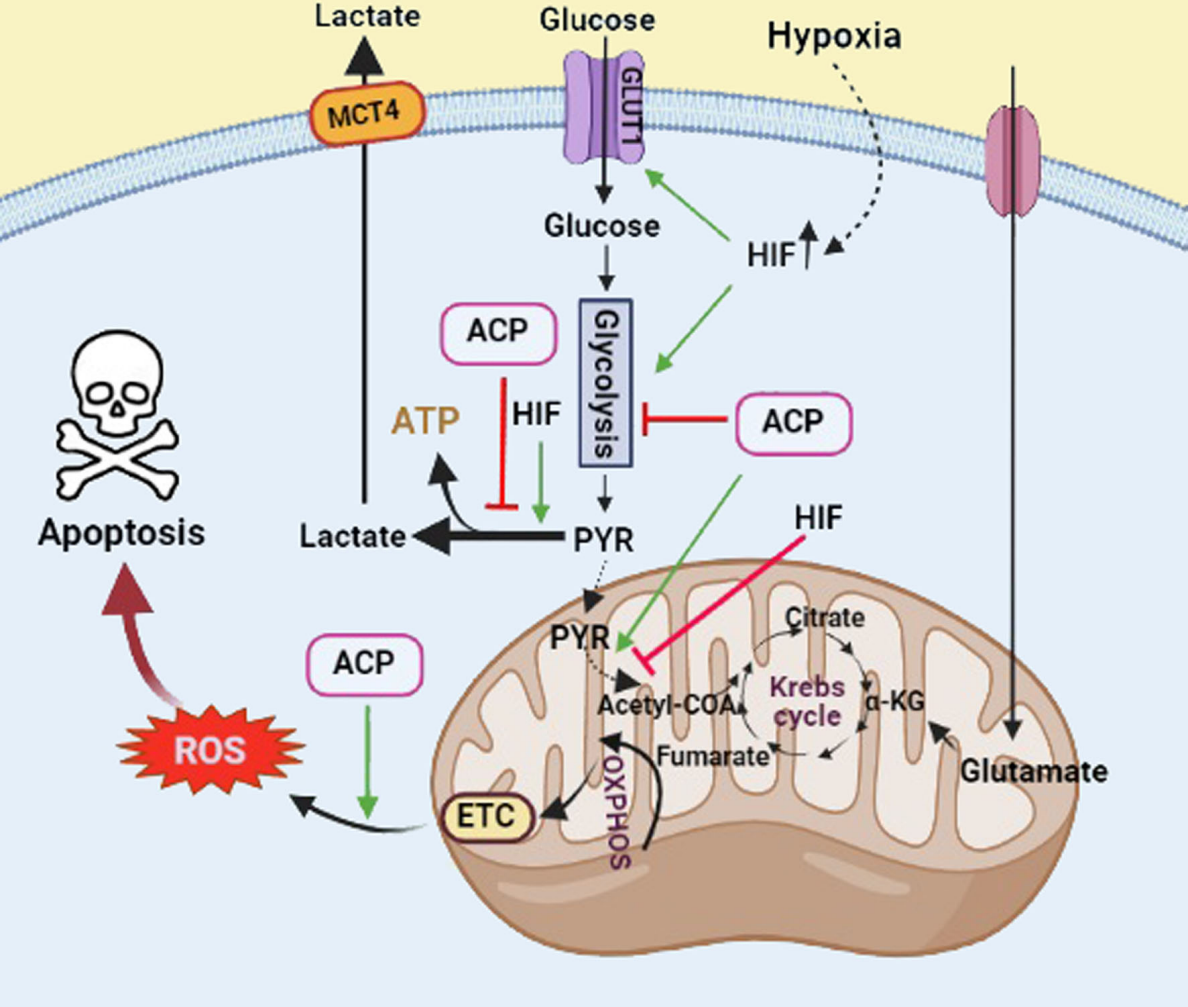
The role of ACPs and HIF in regulation of glucose metabolism in cancer cells. HIF involves the induction of the Warburg effect and glucose transport. ACPs inhibit the glycolysis pathway and induce reactive oxygen species (ROS) production, which leads to cancer cell death through the apoptotic pathway. Black arrows show active glucose metabolic pathways. Green arrows and red T bars show positive and negative impacts on the pathways, respectively. GLUT1, glucose transporter 1; MCT4, monocarboxylate transporter 4 (Created with BioRender.com).

As another mechanism, analysis of the 177 intracellular metabolites and 113 secretory metabolites of colorectal cancer cells showed considerable alteration in their metabolic profile (glycolysis, purine metabolism, and Krebs cycle) in FF/CAP18-treated cells in a dose-dependent manner (117). It is widely acknowledged that hypoxia plays a critical role in cancer metabolism and dormancy that promotes the onset and progression of cancer. Hypoxia is usually present in the TME due to the fast tumor growth without sufficient blood. This condition may affect the release of hypoxia-inducible factor (HIF), which plays a critical role in tumor progression through regulating genes involved in glycolytic metabolism, angiogenesis, and other biological mechanisms. Tumor cells prefer glycolysis to oxidative phosphorylation (OXPHOS) for generating ATP (Warburg effect). As illustrated in **Figure 4**, HIF increases glucose uptake *via* GLUT1 and lactate clearance *via* monocarboxylate transporter (MCT) 4, reprogramming cancer metabolism toward aerobic glycolysis. This altered energy metabolism of tumor cells is ideal for anticancer agents (i.e., ACPs). It is reported that ACPs can disrupt glycolysis and/or mitochondrial respiration to increase ROS production within cancer cells, which leads to cell death through an apoptotic pathway **(** **Figure 5** **)** (117, 118). In this sense, FF/CAP18 reduces the Warburg effect in tumor cells and shifts glucose metabolism toward the pentose phosphate pathway (PPP) (119). One study showed the ability of a synthetic antitumor peptide, CIGB-552, to negatively modulate NF-kB and HIF-1 pathways in human lung cancer cell lines. A proteomic approach performed on CIGB-552-treated cancer cells identified major processes affected by this peptide as oxidative damage, apoptosis, inflammatory response, cell adhesion, and motility (120).

According to some studies, AMPs may display anticancer effects through inducing cell cycle regulatory proteins. For instance, bovine lactoferrin and LfcinB demonstrated proapoptotic activities *via* triggering p53, p21, BRC1, CHK, and ATM pathways. Activation of these pathways mainly results in cell cycle arrest in response to DNA damage (121). In contrast, some studies suggest that AMPs induce their antineoplastic effects independently from cell cycle regulatory signaling pathways. For example, FF/CAP18 yielded antiproliferative effects on human colon cancer cells by depolarizing the mitochondrial membrane. However, the antiproliferative effect of FF/CAP18 was independent of the p53 signaling pathway (122).

MicroRNAs are small noncoding RNAs consisting of 21–25 nucleotides that can suppress messenger RNA (mRNA) translation *via* inducing mRNA degradation. Thus, microRNAs alter gene expression post-transcriptionally regulating mRNA translation (123). It has been observed that LL-37 and its analog peptide, FF/CAP18, upregulated miR-663a in colorectal cancer cells. Upregulated miR-663a directly bound to the coding sequence of chemokine receptor (CXCR) 4 mRNA that resulted in the inhibition of CXCR4 translation and consequent decrease in phosphorylated protein kinase B (Akt). This pathway finally led to p21 activation and tumor cell growth suppression through cell cycle arrest at the G2/M phase (124). In a similar mechanism, KT2 and RT2 derived from *Crocodylus siamensis* caused cell cycle arrest through the upregulation of p21. They also inhibited cancer cell migration *via* the downregulation of PI3K/AKT/mTOR signaling pathways (125).

Combination therapy with AMPs and chemotherapeutic drugs also was evaluated in colorectal cancer. Raileanu et al. assessed the efficiency of Gramicidin A and doxorubicin against the spheroids from colorectal cancer cells. The assessment of cancer cell viability confirmed that both doxorubicin (EC50: 15.31 μg/ml) and Gramicidin A (EC50: 9.78 μM) induce cytotoxic effects against the cancer cell spheroids separately, whereas their combination reduces the cancer cell viability synergistically (5). In another study, 150 μg/ml of KT2 and RT2 inhibited the HCT-116 cell proliferation by 70.65% and 69.01%, respectively. Additionally, the results showed that KT2 and RT2 peptides induced faster growth inhibition (3 h after treatment) on HCT-116 than 5-FU (6 h after treatment), a chemotherapeutic drug used for metastatic colon cancer. Thus, combining KT2 or RT2 peptides with 5-FU may enhance the efficacy of 5-FU (125). As a new drug delivery platform for colorectal cancer therapy, Fan et al. designed a biodegradable and injectable nanoparticle–hydrogel composite of docetaxel and LL37. The designed composite showed inhibitory effects on the growth of colorectal tumors mainly by enhancing apoptosis in neoplastic cells and reducing microvessel density in a colorectal peritoneal carcinomatosis mouse model. By evaluating the platelet endothelial cell adhesion molecule (PECAM-1), mainly found on the endothelial cells, they showed that the nanoparticle–hydrogel composite of docetaxel and LL37 dramatically inhibited angiogenesis in the tumor of the mouse model compared to the free combination of docetaxel and LL37 and pure docetaxel (microvessel density (MVD) was 19.67 ± 3.98 for the nanoparticle–hydrogel composite of docetaxel and LL37, 33.00 ± 7.40 for the free combination of docetaxel and LL37, and 65.50 ± 7.37 for pure docetaxel) (126).

Numerous preclinical *in vitro* studies, as well as insufficient *in vivo* trials, have demonstrated that AMPs either alone or combined with conventional chemotherapy would develop an efficient and safe therapeutic alternative to present chemotherapeutic regimens that are based on the high dose of nonspecific and harmful cytotoxic agents (127). Considering the presence of microbiota in the gastrointestinal tract as a valuable source of AMPs, using engineered microorganisms might improve the prevention and therapeutic role of AMPs in colorectal cancer.

### Glioblastoma

Glioblastoma (GBM) or astrocytoma WHO grade IV is the most aggressive type of primary brain cancer. Despite standard treatments for newly diagnosed patients with GBM, such as total surgical resection, radiotherapy, and temozolomide (TMZ), it has faced the highest mortality rate with a postdiagnostic median survival of 12 to 15 months (128, 129). The low survival rate of GBM can be subjected to the infiltration ability of neoplastic cells into adjacent and/or distant brain tissues and TMZ chemotherapeutic drug resistance.

Studies have shown that AMPs can reduce GBM tumor progression in different manners. Ranatuerin-2PLx (R2PLx), cloned from pickerel frog skin secretions, induced apoptosis in GBM neoplastic cells (103). Guo et al. found two new AMPs by evaluating the cDNA library of the Brazilian yellow scorpion venom, *Tityus serrulatus*. They named these AMPs *Tityus serrulatus* Antimicrobial Peptide (TsAP)1 and TsAP2, showing ineffective and moderate cytotoxicity against glioblastoma cells. It seems that higher helical content and hydrophobic moment in TsAP2 rather than TsAP1 have resulted in more potency of TsAP2. In order to increase the cationicity of these AMPs, they engineered TsAP1 and TsAP2 by replacing residues with neutral side chains with lysyl residues that provided extra positive changes. The intervention enhanced the antineoplastic effect of both TsAP-S1 (EC50 from ineffective to 2.9 mM) and TsAP-S2 (EC50 from 15.4 to 2.0 mM) (130). In another study, dermaseptin-PD-1 and dermaseptin-PD-2, derived from skin secretions of *phyllomedusine* leaf frogs, showed synergistic interaction in inhibiting the proliferation of GBM cancer cell line (131). Epinecidin-1 (epi), an AMP from *Epinephelus coioides*, triggered cytotoxicity in GBM cells by inducing DNA damage and necrosis (132).

Considering complex intracellular signaling pathways in GBM cancer cells, finding the mechanisms of AMP antineoplastic effects in GBM can improve the application of these proteins in therapeutic approaches (133). It has been shown that Tilapia piscidin 3 (TP3) reduced GBM cell migration and metastasis through two different pathways. The first one is preventing rat sarcoma virus (RAS) protein activity by TP3 that subsequently inhibits the phosphorylation of ERK, p38, and JNK. Activation and proper function of these factors are necessary for matrix metalloproteinases 2/9 (MMP2/9) production, which can degrade extracellular matrix (ECM) structural components. ECM can play a crucial role as an anchor to promote cancer cell migration by arranging protein-like focal adhesions (FAs). In other words, TP3 inhibits focal adhesion kinase (FAK), affecting paxillin and reducing FAs (134, 135).

Antimicrobial peptide TP4 induced mitochondrial dysfunction and elevated intracellular ROS production *via* rapidly promoting mitochondrial hyperpolarization. Mitochondrial hyperpolarization mainly results in cell necrosis, whereas apoptosis results from mitochondrial polarization loss. Thus, TP4 displays antineoplastic effects independent of p53 status, bringing them up as adequate antineoplastic protein against both normal and mutant p53 GBM cancer cells (136–138). In addition, LyeTx I-b promotes necrosis or programmed necrosis in GBM cells (139). These necrotic GBM cancer cells release high mobility group box 1 (HMGB1) and cyclophilin A that enhance chemoattraction and maturation of dendritic cells (DCs) as initiators of antitumor immunity (140, 141). Along with inducing ROS production, TP4 reduces antioxidant defenses by suppressing catalase and glutathione peroxidase (GPx) and enhancing the cytotoxic in GBM cells (136).

The combination of AMPs with different antineoplastic agents such as microRNAs and chemotherapeutic drugs has also been considered in studies. Recently, Jana et al. reported that miR210 promotes tumor progression through increasing resistance to TMZ and decreasing the expression of NeuroD2. In their research, Tachyplesin (Tpl) was used as a nanocarrier for anti-miR210 (142). According to the results, P53 induced the transcription of NeuroD2 while miR-210 reduced its amounts post-transcriptionally. NeuroD2 inhibited cell proliferation and migration and promoted apoptosis, weakening GBM aggressiveness (143). In another study, administration of TP4 combined with p38 inhibitors (SB202190 and VX-745) increased the efficacy of GBM treatment (144). Besides the mentioned roles of p38 in GBM progression, this molecule plays a crucial role in GBM chemoresistance due to increasing tumor promoters such as runt-related transcription factor, vascular endothelial growth factor (VEGF), MMP2/9, laminin γ2, nuclear factor erythroid 2-related factor 2 (NRF2), IL-6, and IL-8 (145–148). Meanwhile, TP4 solely upregulates catalase and superoxide dismutase (SOD)1 that reduces the effect of ROS in cancer cell death. However, the combination of TP4 and SB202190 does not upregulate SOD1 that obviously induces ROS generation (144).

Considering complex intracellular pathways of colorectal cancer cells, AMPs have induced their antineoplastic effects not only by the conventional disrupting cell membrane but also by interfering with critical signalings such as ERK, p38, JNK, and FAK (134, 135). Besides, TP4 has shown promising anticancer effects independent of p53 status, making this AMP more appropriate for treating GBM cancer cells with impaired p53 mutations. A new combination therapy insight in colorectal cancer uses microRNAs with AMPs like TP4 (142). This therapeutic approach will help the translation of epigenetic alteration combined with AMP application in cancer treatment.

### Lung Cancer

Lung cancer, including small-cell lung cancer (SCLC) and nonsmall-cell lung carcinoma (NSCLC), is one of the most important leading causes of cancer deaths worldwide, with a 5-year survival rate of 20% (149). The 5-year survival rate for NSCLC patients, especially at advanced stages III and IV, is about 15–20% (www.cancer.net). Similar to other cancers, the therapeutic strategies consist of surgery, chemotherapy, radiotherapy, targeted therapy, immunotherapy, and palliative therapy (150, 151).

AMPs have shown promising antineoplastic features such as cytotoxicity, antiproliferation, reducing cell adhesion, interfering with tubulins, and anti-angiogenesis in preclinical studies of lung cancer treatment. Bombinin H4 (100–1.5 μM) and temporin A (100–50 μM) induced cytotoxic effects in lung cancer cells. Additionally, theses AMPs showed low hemolytic activity compared to untreated cells that were 0.061% and 0.874% for bombinin H4 and temporin A, respectively (152). Phylloseptin-PC (PSN-PC), derived from the skin secretion of the *Phyllomedusa camba*, displayed an antiproliferation effect on the NSCLC cell line (NCI-H157) (153). Liu and coworkers introduced two novel Phylloseptin: phylloseptin-PTa and phylloseptin-PHa, which inhibited the proliferation of the NSCLC line H157 (154). CB1a, an engineered modification of Cecropin B, induced cytotoxicity in lung cancer cells at nontoxic concentrations for normal lung cells in which the EC50 was 29 ± 4.3 for NSCLC and 4 ± 0.6 for SCLC. Also, CB1a prevented cell–cell adhesion, inhibited tumor-like spheroid formation, and significantly suppressed the growth of human lung tumor xenograft model (155).

Based on the evidence, TP4 can induce microtubule disruption and destabilization through specific interactions between TP4 and tubulins. There are crucial residues in TP4 required for the antineoplastic interaction of TP4 and tubulin, including Phe1 or Ile16 and Arg23 (156). It seems that microtubule disruption is a result of FosB function. Activation of FosB induces two significant antineoplastic effects; first, FosB disturbs cytoskeletal and membrane consistency in NSCLC cells. Second, FosB activates protocadherin beta-13 (PCDHB13), which controls and disrupts the microtubule dynamics. It correlates with clinical observation in which both FosB and PCDHB13 expression were negatively associated with the clinical course of patients with NSCLC (107, 157).

AMPs also have anti-angiogenic effects in lung cancer. It has been indicated that LfcinB suppressed the transcription and translation of the VEGF gene in H460 cells. LfcinB correspondingly increased the ROS generation and suppressed the translation of antioxidant enzymes such as glutathione peroxidase (GPX) 1/2, SOD3, and catalase. It also reduced the growth of lung tumors in animal models (158).

The growing studies evaluating the effects of AMPs on lung cancer have improved our understanding of the anticancer role of AMPs in lung cancer. Preclinical results have shown the cytotoxic, antiproliferative, and anti-angiogenic effects of AMPs in lung cancer. However, more studies are required to shed light on the other anticancer mechanisms as well as possible combination therapeutic approaches.

### Hematologic Malignancies

Hematologic malignancies are mainly caused by neoplastic effects in the blood, bone marrow, and lymph nodes. The main types of leukemia are acute myeloid (AML), chronic myeloid (CML), acute lymphocytic (ALL), and chronic lymphocytic (CLL). It also comprises myeloma and two types of lymphoma: Hodgkin’s and non-Hodgkin’s (NHL) (159). Hematologic malignancies have reduced the quality of life, especially in developing and emerging countries (160).

AMPs have displayed considerable therapeutic effects in hematologic malignancies. Considering various hematologic malignancies, shedding light on the underlying mechanisms of AMPs’ antineoplastic effects will improve our understanding of new therapeutic methods. It has been shown that AMPs induce antineoplastic effects through cell membrane disruption, DNA damage, apoptosis-inducing factor (AIF), and calcium leakage from the endoplasmic reticulum (ER). Smp24 and Smp43, novel AMPs derived from the venom of the Egyptian scorpion (*Scorpio maurus palmatus*), reduced the cell viability of acute myeloid and lymphoid leukemia cell lines (161). Evaluation of NRC-03 and NRC-07 cytotoxicity in multiple myeloma cells showed considerable cell death mainly through extensive membrane damage and DNA cleavage. Besides, intratumoral administration of NRC-03 reduced the progression of multiple myeloma xenografts in immune-deficient mice models (162). Jurkat T leukemia cells generated ROS followed by caspase-2-induced impairment of mitochondrial membrane potential as well as activation of caspase-9 and caspase-3 after treatment with LfcinB (163).

LL-37 has been shown to induce apoptosis through a mitochondria-related pathway, independent of caspase and dependent on AIF. Exposure to LL-37 triggered the translocation of AIF from cell mitochondria to the nucleus, where it activates chromatin condensation and DNA fragmentation that results in programmed cell death (164, 165). It was demonstrated that AMP-induced intracellular calcium leakage could trigger cancer cell death. Yudie Lv et al. showed that PFR, a sort of AMP identified from the derivatives of lactoferrin, could promote necroptosis *via* ER stress and consequent elevated cytoplasmic calcium. They disclosed that raised cytoplasmic calcium resulted from both the influx of extracellular calcium and the release of intracellular calcium from ER due to ER stress. In addition, it was indicated that mitochondrial calcium played no role in the cytotoxicity of PFR in AML cells (166).

Administrating combination therapy and using AMP-based vaccines have been studied in the area of hematologic malignancies. Cytosine arabinoside (Ara-C) is a standard chemotherapeutic drug used as a pharmacologic regime in AML patients. Developing drug resistance and considerable side effects are critical challenges in using Ara-C (167, 168). It was observed that the combination of PFR and Ara-C significantly suppressed the AML xenograft tumor growth in a mouse model. Besides, there were no considerable general side effects or toxicity in the liver and kidney (166).

Berge G and colleagues attempted to design an AMP-based vaccine for B cell lymphomas. They injected LTX-302 (0.5 mg LTX-302/50 μl saline) into the tumors of the animal cancer model, which led to tumor necrosis and inflammatory cell infiltration. The tumor rechallenge test showed specific protection against B-cell lymphomas, which was both CD4^+^ and CD8^+^ T-cell dependent (169). The mechanism of LTX-302-induced immunity seemed to reflect two exclusive mechanisms. The first is that cell lysis and following release of the intracellular content to the extracellular space could increase the amount of tumor-associated antigen that stimulated T cells through antigen-presenting cells (APCs) (170). The second is that resultant danger signals from tumor necrosis could enhance the maturation and performance of DCs that result in T-cell activation (171).

A novel approach to using AMPs in cancer treatment is designing vaccines. Besides the common antineoplastic features of AMPs, which can be seen in various cancer types, utilizing AMP-based vaccines against cancer may improve cancer prevention and provide hopeful prospects in cancer treatment.

## Clinical Applications of AMPs in Anticancer Therapy

Following preclinical assessments, AMPs have entered clinical trials and clinical practice. From the antimicrobial point of view, AMPs have been widely studied in several clinical trials, and also there are several ongoing clinical trials on www.clinicaltrials.gov (**Table 2**). Besides, many AMPs such as hLF1–11, pexiganan acetate, CZEN-002, omiganan, and novexatin have obtained U.S. Food and Drug Administration (FDA) approval for clinical use for treating various infectious diseases (172–175). In this regard, pexiganan acetate, a synthetic analogue of magainin 2 with 22 amino acids, was the first commercially developed AMP that has been used in infected diabetic foot ulcers (173). Along with topical administration, the safety of intravenous injection of some AMPs was evaluated. In this regard, the safety and tolerability of human lactoferrin 1-11 (hLF1-11) were assessed in the life-threatening infections of patients with hematological malignancies who received hematopoietic stem cell transplantation (HSCT). The results showed that patients could tolerate up to 5-mg single dose of hLF1-11 intravenously (174).

**Table 2:** Clinical trials of AMP administration in infectious diseases.

Phase	AMP type	Pathologic condition	Route of administration	Primary Purpose	NCT number
1	TAPS-18	Periodontitis	Topical	Treatment	NCT05125718
2	C16G2	Dental Caries	Topical	Prevention	NCT02509845
	C16G2	Dental Caries	Topical	Treatment	NCT03004365
	MBI 226	Acne Vulgaris	Topical	Treatment	NCT00211523
	C16G2	Dental Caries	Topical	Treatment	NCT03196219
	C16G2	Dental Caries	Topical	Prevention	NCT02254993
	C16G2	Dental Caries	Topical	Treatment	NCT02594254
	NVXT	Onychomycosis	Topical	Treatment	NCT02933879
	PAC113	Oral Candidiasis	Topical	Treatment	NCT00659971
	Dalbavancin	Osteomyelitis	Intravenous	Treatment	NCT02685033
	Brilacidin	Skin Infections	Topical	Treatment	NCT02052388
	AB103	Necrotizing Soft Tissue Infections	Topical	Treatment	NCT01417780
3	Pexiganan	Diabetic Foot Infection	Topical	Treatment	NCT01594762
	CB-183,315	*Clostridium difficile* Infection	Oral	Treatment	NCT01597505

AMPs have recently been introduced to the field of anticancer therapy; thus, safety and efficacy data are pretty limited. However, some ongoing or completed clinical trials investigated the antineoplastic effects of AMPs (www.clinicaltrials.gov). A recently completed clinical trial (NCT02225366) has evaluated the appropriate dose of LL37 that can be administered intratumorally in patients with melanoma. In this regard, tumor sizes were measured and photographed one week prior to administrating LL37 and again after four weeks. Additionally, the antineoplastic effects of LL37 on the immune response, especially T-cell activity and interferon-alpha expression, were evaluated. In this regard, a phase I multicentral study (NCT01058616) aimed to assess the safety profile and immunological response of LTX-315 (Oncopore™) administered in patients with transdermal accessible tumors. Studies also have assessed the antineoplastic effects of the combination of AMPs with chemotherapeutic agents. Sarina Piha-Paul et al. administered an intravenous infusion of LTX-315 in combination with pembrolizumab, an immune checkpoint inhibitor, in melanoma and TNBC (NCT04796194).

Wilm’s Tumor 1 (WT1) peptide is overexpressed in many malignancies and solid cancers. A phase 1/2 study investigated the effect of DSP-7888, a novel WT1-based peptide vaccine, in patients with myelodysplastic syndrome (MDS). The finding of the study indicated that DSP-7888 is well tolerated in MDS patients, although injection site reaction was observed in all patients (176). A phase 2 clinical trial (NCT00433745) was launched to assess the safety and immunogenicity of a combined vaccine of 2 leukemia-associated antigenic peptides, PR1 and WT1, for patients with high-risk hematological malignancies. In this study, nine doses of WT-1 were administered concomitantly with GM-CSF (Sargramostim). Some parameters, including the numbers of blood cells expressing WT1, decrease in bone marrow blast cells, alteration in blood counts, dependence to transfusion, time to disease progression, and survival rate, were assessed as disease response indicators. Seven weeks after the last vaccination, the possible side effects and circulating WT1-specific T-cell number were evaluated. The results showed that this combined vaccine approach is safe and can elicit immunologic responses associated with a reduction in WT1 expression in patients.

Although studies are evaluating the possibility of AMP translation into clinical practice, there is still a lack of strong supporting evidence of administrating AMPs in patients with cancer. In other words, finding optimal doses and routes of AMP administration to achieve the desired therapeutic outcomes with the lowest toxicity and proposing appropriate technical methods to improve their specificity, stability, and pharmacokinetics are considered essential questions that should be answered in future studies.

## The Challenges and Prospects of Anticancer Peptides in Clinical Applications

Many advantages of AMPs/ACPs, including broad-spectrum activities, rapid onset of activities, low toxicity, and relatively low risk of resistance, have made them attractive candidates in clinical treatments. Although there is hope for translating AMPs into clinical practice, some obstacles, including collateral toxicity on normal mammalian cells, short half-life, severe immune reaction, and high cost of production, impede the clinical use of these antineoplastic agents. Some studies have reported that long-term AMP administration can exert considerable collateral toxicity on normal mammalian cells (177). Besides, some reports of hemolytic activity of some AMPs such as indolicidin and melittin prevented clinical use (178). It seems that more lipophilic peptides and also the presence of some amino acids, including tryptophan, lysine, and arginine, tend to be more hemolytic (179). In order to surmount these problems, some studies attempted to design AMPs’ analogs. Staubitz et al. altered all of the five tryptophan residues of indolicidin to phenylalanine that reduced the hemolytic activity, while the antimicrobial activity against *M. luteus* remained intact (180). M.P. Smirnova et al. synthesized 45 analogs of indolicidin, among which the most efficient showed at least 1.8 times lower hemolytic activity compared to indolicidin (181). As another concern, poor pharmacokinetics described as short half-life, fast elimination, instability, enzymatic degradation, poor distribution by binding to serum proteins, and poor GI absorption reduced the chance of clinical translation of AMPs and drug development pipelines (127).

The redesigning and modification of AMPs results in preserving their advantages simultaneous to reducing their collateral toxicities, thereby eliminating the aforementioned bottlenecks and improving the antineoplastic activity of AMPs. The design of an AMP with optimized characteristics and high impact on the cancer treatment area requires accurate information about the activity of peptides on the cell membranes. Therefore, obtaining the optimal AMP depends on manipulating some parameters such as its sequence, secondary structure, net charge, and hydrophobicity (182). Two main strategies can describe the reconstruction of AMPs: 1) main-chain alteration that is explained as replacing natural or non-natural amino acids, and 2) side-chain alteration that primarily includes phosphorylation, polyethylene glycol modification, cholesterol modification, glycosylation, and palmitoylation (183). The FDA-approved AMPs, e.g., Colistin, Vancomycin, Oritavancin, and Dalbavancin, are small, with a molecular weight range from 1,155 to 1,817. They have elimination half-lives of 5 h, 7.5 h, 195.4 h, and 14 days, respectively (184). Properties such as having several unconventional amino acids and chemical changes or cyclic structures have optimized the pharmacokinetics and resistance of these peptides to enzymatic degradation.

The severe immune response is an additional obstacle to the use of ACPs in cancer treatment. Introducing external ACPs into the host can induce therapeutically neutralizing antibodies and/or elicit harmful allergic reactions in cancer patients. Therefore, several strategies have been considered to overcome this drawback. To prevent harmful anti-ACP immune responses, the introduction of host defense peptides (HDPs) or co-administration of foreign ACP with immunosuppressants may be a palliative strategy. Besides, encapsulation of ACP in liposomes designed to deliver cargo directly to the tumor site may be promising because it minimizes the chances of the host acquiring anti-ACP immunity (185).

Utilizing chemical delivery vehicles is another approach to improving these molecules’ stability, half-life, therapeutic properties, and activity. Inorganic nanomaterials are among the candidates for designing delivery platforms. These substances protect AMPs from chemical and enzymatic digestion, regulate drug release rate, enhance the bioavailability, and diminish toxicity. Betides, these substances efficiently interact with external factors such as magnetic field or light that can trigger drug release or localize drug accumulation (186, 187). Polymeric materials are also used in AMP delivery platforms that can be designed to synergistically cooperate with AMP to induce antineoplastic effects (188). As another point of view, using biological carriers such as exosomes, nanosized biovesicles can protect AMPs from external disturbances and also provide enhanced efficacy by adding their antineoplastic effects. Exosomes escape from immune rejection, work in a targeted manner toward tumors, and are easily isolated from original cells (68).

Collectively, finding an ideal peptide and overcoming the drug limitations of these exciting molecules is a great and promising opportunity for their clinical applications shortly.

## Computational Approaches in AMP Design Against Cancers

Computational biology is a rapidly progressing multidisciplinary field (189). In addition to experimental approaches, computational and bioinformatics strategies play an essential role in peptide design and cancer therapeutics. Various challenges of practical techniques, such as experimental manpower, time, and environmental and safety issues, can be partially addressed by computational methods (151). In the context of AMP design, advanced rational design strategies have been used in combination with computational approaches to develop more robust and economical AMPs. The design of an AMP with optimized characteristics and high impact on the cancer treatment area requires accurate information about the anticancer activity of peptides. The *de novo* computational method uses amino acid frequency and position priorities to predict and generate low-cost AMP sequences. Given the rapid development of computational tools in recent years, these features will certainly assist an increasing number of computationally designed AMPs to evolve from database sequences to real, effective drug candidates that are more likely to reach the market in upcoming years. A computational method will help reduce production costs *via* identifying the amino acid sequence in the complete sequence of peptides that may be responsible for anticancer activity by synthesizing shorter biologically active fragments. Therefore, obtaining the optimal AMP depends on manipulating some parameters such as its sequence, secondary structure, net charge, and hydrophobicity (182, 190). In addition, many pharmacological parameters such as bioavailability, stability, and even immunogenicity will be improved (190).

Currently, several computational tools have been established to design AMP variants, including empirical methods, machine learning, and stochastic approaches (191). For instance, an anticancer scanner (ACPS) has been demonstrated an effective software package based on a nature-encouraged algorithm (ANN) to consider anticancer target clinical information and recognize possibly potent peptides. Tools like this are helpful for clinicians and researchers in the field of oncology to employ in personalized medicine exploration and structure-based drug design.

## Conclusions

The urgent need to obtain new agents to prevent and treat cancers has been driving the ACP research. In this light, ACPs are considered promising agents for producing new anticancer drugs and vaccines. Some ACPs have been demonstrated to be antiproliferative and proapoptotic in numerous cancer cell types, both *in vitro *and *in vivo*, leading to clinical trials evaluating cancer treatment addressed in the present review. None of the current known AMP-based trends are exempt from the obstacles. In this regard, the continued development of computational methods that helped better understand the mechanism of action of ACP, including the discovery of new molecular targets, will significantly influence the design of new ACP agents.

Although many challenges to clinical applications need to be overcome, natural and synthetic AMPs remain attractive sources for pharmaceutical companies. Proteomic/genomic technologies combined with data mining, ACP prediction, and virtual screening can be beneficial in facilitating the commercial development of anticancer peptides.

Taken together, ACPs could herald the emergence of a novel strategy for developing anticancer drugs or vaccines to reduce new cases and mortality rates in the future.

## Author Contributions

Conceptualization, AJ and AB. Methodology, AJ and RSF. Investigation, AJ, AB, and MY. Writing—original draft preparation, AJ and AB. Critically revision and editing, AJ, AB, and MY. Visualization, RSF and MR-T. Supervision, MR-T. Project administration, AJ and MR-T. Funding acquisition, MR-T. All authors read and approved the final manuscript.

## Conflict of Interest

The authors declare that the research was conducted in the absence of any commercial or financial relationships that could be construed as a potential conflict of interest.

## Publisher’s Note

All claims expressed in this article are solely those of the authors and do not necessarily represent those of their affiliated organizations, or those of the publisher, the editors and the reviewers. Any product that may be evaluated in this article, or claim that may be made by its manufacturer, is not guaranteed or endorsed by the publisher.

## References

[1] Bhatia S Frangioni JV Hoffman RM Iafrate AJ Polyak K. The Challenges Posed by Cancer Heterogeneity. Nat Biotechnol (2012) 30(7):604–10. doi: 10.1038/nbt.2294 22781679

[2] Jafari A Rezaei-Tavirani M Salimi M Tavakkol R Jafari Z. Oncological Emergencies From Pathophysiology and Diagnosis to Treatment: A Narrative Review. Soc Work Public Health (2020) 35(8):689–709. doi: 10.1080/19371918.2020.1824844 32967589

[3] Fernandes C Costa A Osório L Lago RC Linhares P Carvalho B. “Current Standards of Care in Glioblastoma Therapy.” In *Glioblastoma*, ed. De Vleeschouwer S. Brisbane (AU): Codon Publications (2017) p. 197–241. 29251860

[4] Abbas Z Rehman S. An Overview of Cancer Treatment Modalities. Neoplasm (2018) 1:139–57. doi: 10.5772/intechopen.76558

[5] Raileanu M Popescu A Bacalum M. Antimicrobial Peptides as New Combination Agents in Cancer Therapeutics: A Promising Protocol Against HT-29 Tumoral Spheroids. Int J Mol Sci (2020) 21(18):6964. doi: 10.3390/ijms21186964 PMC7555805 32971958

[6] Gaspar D Freire JM Pacheco TR Barata JT Castanho MA. Apoptotic Human Neutrophil Peptide-1 Anti-Tumor Activity Revealed by Cellular Biomechanics. Biochim Biophys Acta (BBA)-Molecular Cell Res (2015) 1853(2):308–16. doi: 10.1016/j.bbamcr.2014.11.006 25447543

[7] Zhong L Li Y Xiong L Wang W Wu M Yuan T. Small Molecules in Targeted Cancer Therapy: Advances, Challenges, and Future Perspectives. Signal Transduct Target Ther (2021) 6(1):1–48. doi: 10.1038/s41392-021-00572-w 34054126 PMC8165101

[8] Zhang C Yang M Ericsson AC. Antimicrobial Peptides: Potential Application in Liver Cancer. Front Microbiol (2019) 10:1257. doi: 10.3389/fmicb.2019.01257 31231341 PMC6560174

[9] Thapa RK Diep DB Tønnesen HH. Topical Antimicrobial Peptide Formulations for Wound Healing: Current Developments and Future Prospects. Acta Biomater (2020) 103:52–67. doi: 10.1016/j.actbio.2019.12.025 31874224

[10] Kawano Y Jordan O Hanawa T Borchard G Patrulea V. Are Antimicrobial Peptide Dendrimers an Escape From ESKAPE? Adv Wound Care (New Rochelle) (2020) 9(7):378–95. doi: 10.1089/wound.2019.1113 PMC7307686 32320368

[11] Ye G Wu H Huang J Wang W Ge K Li G. LAMP2: A Major Update of the Database Linking Antimicrobial Peptides. Database (2020) 2020:1–6. doi: 10.1093/database/baaa061 32844169 PMC7447557

[12] Haney EF Mansour SC Hilchie AL de la Fuente-Núñez C Hancock RE. High Throughput Screening Methods for Assessing Antibiofilm and Immunomodulatory Activities of Synthetic Peptides. Peptides (2015) 71:276–85. doi: 10.1016/j.peptides.2015.03.015 PMC4581888 25836992

[13] Tonk M Vilcinskas A Rahnamaeian M. Insect Antimicrobial Peptides: Potential Tools for the Prevention of Skin Cancer. Appl Microbiol Biotechnol (2016) 100(17):7397–405. doi: 10.1007/s00253-016-7718-y PMC4980408 27418360

[14] Dennison SR Whittaker M Harris F Phoenix DA. Anticancer α-Helical Peptides and Structure/Function Relationships Underpinning Their Interactions With Tumour Cell Membranes. Curr Protein Pept Sci (2006) 7(6):487–99. doi: 10.2174/138920306779025611 17168782

[15] Deslouches B Di YP. Antimicrobial Peptides With Selective Antitumor Mechanisms: Prospect for Anticancer Applications. Oncotarget (2017) 8(28):46635. doi: 10.18632/oncotarget.16743 28422728 PMC5542299

[16] Riedl S Zweytick D Lohner K. Membrane-Active Host Defense Peptides–Challenges and Perspectives for the Development of Novel Anticancer Drugs. Chem Phys Lipids (2011) 164(8):766–81. doi: 10.1016/j.chemphyslip.2011.09.004 PMC3220766 21945565

[17] Domalaon R Findlay B Ogunsina M Arthur G Schweizer F. Ultrashort Cationic Lipopeptides and Lipopeptoids: Evaluation and Mechanistic Insights Against Epithelial Cancer Cells. Peptides (2016) 84:58–67. doi: 10.1016/j.peptides.2016.07.007 27486068

[18] Figueiredo CR Matsuo AL Massaoka MH Polonelli L Travassos LR. Anti-Tumor Activities of Peptides Corresponding to Conserved Complementary Determining Regions From Different Immunoglobulins. Peptides (2014) 59:14–9. doi: 10.1016/j.peptides.2014.06.007 24972300

[19] Wu D Gao Y Qi Y Chen L Ma Y Li Y. Peptide-Based Cancer Therapy: Opportunity and Challenge. Cancer Lett (2014) 351(1):13–22. doi: 10.1016/j.canlet.2014.05.002 24836189

[20] Lei J Sun L Huang S Zhu C Li P He J. The Antimicrobial Peptides and Their Potential Clinical Applications. Am J Trans Res (2019) 11(7):3919. PMC6684887 31396309

[21] Available at: https://aps.unmc.edu/AP.

[22] Kościuczuk EM Lisowski P Jarczak J Strzałkowska N Jóźwik A Horbańczuk J. Cathelicidins: Family of Antimicrobial Peptides. A Review. Mol Biol Rep (2012) 39(12):10957–70. doi: 10.1007/s11033-012-1997-x PMC3487008 23065264

[23] Huan Y Kong Q Mou H Yi H. Antimicrobial Peptides: Classification, Design, Application and Research Progress in Multiple Fields. Front Microbiol (2020) 11:2559. doi: 10.3389/fmicb.2020.582779 PMC7596191 33178164

[24] Kumar R Ali SA Singh SK Bhushan V Mathur M Jamwal S. Antimicrobial Peptides in Farm Animals: An Updated Review on Its Diversity, Function, Modes of Action and Therapeutic Prospects. Vet Sci (2020) 7(4):206. doi: 10.3390/vetsci7040206 PMC7766339 33352919

[25] Agrawal S Acharya D Adholeya A Barrow CJ Deshmukh SK. Nonribosomal Peptides From Marine Microbes and Their Antimicrobial and Anticancer Potential. Front Pharmacol (2017) 8:828. doi: 10.3389/fphar.2017.00828 29209209 PMC5702503

[26] Ashby M Petkova A Gani J Mikut R Hilpert K. Use of Peptide Libraries for Identification and Optimization of Novel Antimicrobial Peptides. Curr topics Med Chem (2017) 17(5):537–53. doi: 10.2174/1568026616666160713125555 27411326

[27] Sudheendra U Dhople V Datta A Kar RK Shelburne CE Bhunia A. Membrane Disruptive Antimicrobial Activities of Human β-Defensin-3 Analogs. Eur J med Chem (2015) 91:91–9. doi: 10.1016/j.ejmech.2014.08.021 PMC4310808 25112689

[28] Li Y Xiang Q Zhang Q Huang Y Su Z. Overview on the Recent Study of Antimicrobial Peptides: Origins, Functions, Relative Mechanisms and Application. Peptides (2012) 37(2):207–15. doi: 10.1016/j.peptides.2012.07.001 22800692

[29] Lee H-T Lee C-C Yang J-R Lai JZ Chang KY. A Large-Scale Structural Classification of Antimicrobial Peptides. BioMed Res Int (2015) 2015:1–6. doi: 10.1155/2015/475062 PMC4426897 26000295

[30] Zelezetsky I Tossi A. Alpha-Helical Antimicrobial Peptides—Using a Sequence Template to Guide Structure–Activity Relationship Studies. Biochim Biophys Acta (BBA)-Biomembr (2006) 1758(9):1436–49. doi: 10.1016/j.bbamem.2006.03.021 16678118

[31] Marsh D Jost M Peggion C Toniolo C. Lipid Chain-Length Dependence for Incorporation of Alamethicin in Membranes: Electron Paramagnetic Resonance Studies on TOAC-Spin Labeled Analogs. Biophys J (2007) 92(11):4002–11. doi: 10.1529/biophysj.107.104026 PMC1868974 17351010

[32] Veldhuizen EJ Schneider VA Agustiandari H Van Dijk A Tjeerdsma-van Bokhoven JL Bikker FJ. Antimicrobial and Immunomodulatory Activities of PR-39 Derived Peptides. PloS One (2014) 9(4):e95939. doi: 10.1371/journal.pone.0095939 24755622 PMC3995882

[33] Xie M Liu D Yang Y. Anti-Cancer Peptides: Classification, Mechanism of Action, Reconstruction and Modification. Open Biol (2020) 10(7):200004. doi: 10.1098/rsob.200004 32692959 PMC7574553

[34] Lehmann J Retz M Sidhu SS Suttmann H Sell M Paulsen F. Antitumor Activity of the Antimicrobial Peptide Magainin II Against Bladder Cancer Cell Lines. Eur Urol (2006) 50(1):141–7. doi: 10.1016/j.eururo.2005.12.043 16476519

[35] Anghel R Jitaru D Bădescu L Bădescu M Ciocoiu M. The Cytotoxic Effect of Magainin II on the MDA-MB-231 and M14K Tumour Cell Lines. BioMed Res Int (2013) 2013:1–11. doi: 10.1155/2013/831709 PMC3809593 24222919

[36] Rozek T Wegener KL Bowie JH Olver IN Carver JA Wallace JC. The Antibiotic and Anticancer Active Aurein Peptides From the Australian Bell Frogs Litoria Aurea and Litoria Raniformis the Solution Structure of Aurein 1.2. Eur J Biochem (2000) 267(17):5330–41. doi: 10.1046/j.1432-1327.2000.01536.x 10951191

[37] Lee HS Park CB Kim JM Jang SA Park IY Kim MS. Mechanism of Anticancer Activity of Buforin IIb, a Histone H2A-Derived Peptide. Cancer Lett (2008) 271(1):47–55. doi: 10.1016/j.canlet.2008.05.041 18617323

[38] Wang C Dong S Zhang L Zhao Y Huang L Gong X. Cell Surface Binding, Uptaking and Anticancer Activity of L-K6, a Lysine/Leucine-Rich Peptide, on Human Breast Cancer MCF-7 Cells. Sci Rep (2017) 7(1):1–13. doi: 10.1038/s41598-017-08963-2 28811617 PMC5557901

[39] Ren SX Shen J Cheng AS Lu L Chan RL Li ZJ. FK-16 Derived From the Anticancer Peptide LL-37 Induces Caspase-Independent Apoptosis and Autophagic Cell Death in Colon Cancer Cells. PloS One (2013) 8(5):e63641. doi: 10.1371/journal.pone.0063641 23700428 PMC3659029

[40] Ghavami S Asoodeh A Klonisch T Halayko AJ Kadkhoda K Kroczak TJ. Brevinin-2R1 Semi-Selectively Kills Cancer Cells by a Distinct Mechanism, Which Involves the Lysosomal-Mitochondrial Death Pathway. J Cell Mol Med (2008) 12(3):1005–22. doi: 10.1111/j.1582-4934.2008.00129.x PMC4401144 18494941

[41] Wang K-R Zhang B-Z Zhang W Yan J-X Li J Wang R. Antitumor Effects, Cell Selectivity and Structure–Activity Relationship of a Novel Antimicrobial Peptide Polybia-MPI. Peptides (2008) 29(6):963–8. doi: 10.1016/j.peptides.2008.01.015 18328599

[42] Dos Santos C Hamadat S Le Saux K Newton C Mazouni M Zargarian L. Studies of the Antitumor Mechanism of Action of Dermaseptin B2, a Multifunctional Cationic Antimicrobial Peptide, Reveal a Partial Implication of Cell Surface Glycosaminoglycans. PloS One (2017) 12(8):e0182926. doi: 10.1371/journal.pone.0182926 28797092 PMC5552233

[43] Xu X Jiang H Li H Zhang T Zhou Q Liu N. Apoptosis of Stomach Cancer Cell SGC-7901 and Regulation of Akt Signaling Way Induced by Bovine Lactoferrin. J dairy Sci (2010) 93(6):2344–50. doi: 10.3168/jds.2009-2926 20494139

[44] Hilchie AL Vale R Zemlak TS Hoskin DW. Generation of a Hematologic Malignancy-Selective Membranolytic Peptide From the Antimicrobial Core (RRWQWR) of Bovine Lactoferricin. Exp Mol Pathol (2013) 95(2):192–8. doi: 10.1016/j.yexmp.2013.07.006 23892223

[45] Meng L Xu G Li J Liu W Jia W Ma J. Bovine Lactoferricin P13 Triggers ROS−Mediated Caspase−Dependent Apoptosis in SMMC7721 Cells. Oncol Lett (2017) 13(1):511–7. doi: 10.3892/ol.2016.5415 PMC5244845 28123590

[46] Ryu M-J Anikin V Hong S-H Jeon H Yu YG Yu M-H. Activation of NF-κb by Alloferon Through Down-Regulation of Antioxidant Proteins and Iκbα. Mol Cell Biochem (2008) 313(1):91–102. doi: 10.1007/s11010-008-9746-0 18363038

[47] Hu E Wang D Chen J Tao X. Novel Cyclotides From Hedyotis Diffusa Induce Apoptosis and Inhibit Proliferation and Migration of Prostate Cancer Cells. Int J Clin Exp Med (2015) 8(3):4059. 26064310 PMC4443144

[48] Zhang G Liu S Liu Y Wang F Ren J Gu J. A Novel Cyclic Pentapeptide, H−10, Inhibits B16 Cancer Cell Growth and Induces Cell Apoptosis. Oncol Lett (2014) 8(1):248–52. doi: 10.3892/ol.2014.2121 PMC4063637 24959255

[49] Wang Y Guo D He J Song L Chen H Zhang Z. Inhibition of Fatty Acid Synthesis Arrests Colorectal Neoplasm Growth and Metastasis: Anti-Cancer Therapeutical Effects of Natural Cyclopeptide RA-XII. Biochem Biophys Res Commun (2019) 512(4):819–24. doi: 10.1016/j.bbrc.2019.03.088 30928092

[50] Haney EF Straus SK Hancock RE. Reassessing the Host Defense Peptide Landscape. Front Chem (2019) 7:43. doi: 10.3389/fchem.2019.00043 30778385 PMC6369191

[51] Mahlapuu M Håkansson J Ringstad L Björn C. Antimicrobial Peptides: An Emerging Category of Therapeutic Agents. Front Cell infect Microbiol (2016) 6:194. doi: 10.3389/fcimb.2016.00194 28083516 PMC5186781

[52] Rathinakumar R Wimley WC. High-Throughput Discovery of Broad-Spectrum Peptide Antibiotics. FASEB J (2010) 24(9):3232–8. doi: 10.1096/fj.10-157040 PMC2923358 20410445

[53] Yang M Zhang C Zhang MZ Zhang S. Novel Synthetic Analogues of Avian β-Defensin-12: The Role of Charge, Hydrophobicity, and Disulfide Bridges in Biological Functions. BMC Microbiol (2017) 17(1):1–14. doi: 10.1186/s12866-017-0959-9 28231771 PMC5324278

[54] Piotrowska U Sobczak M Oledzka E. Current State of a Dual Behaviour of Antimicrobial Peptides—Therapeutic Agents and Promising Delivery Vectors. Chem Biol Drug design (2017) 90(6):1079–93. doi: 10.1111/cbdd.13031 28548370

[55] Roudi R Syn NL Roudbary M. Antimicrobial Peptides as Biologic and Immunotherapeutic Agents Against Cancer: A Comprehensive Overview. Front Immunol (2017) 8:1320. doi: 10.3389/fimmu.2017.01320 29081781 PMC5645638

[56] Lee TH Hall KN Aguilar MI. Antimicrobial Peptide Structure and Mechanism of Action: A Focus on the Role of Membrane Structure. Curr Top Med Chem (2016) 16(1):25–39. doi: 10.2174/1568026615666150703121700 26139112

[57] Kumar P Kizhakkedathu JN Straus SK. Antimicrobial Peptides: Diversity, Mechanism of Action and Strategies to Improve the Activity and Biocompatibility In Vivo. Biomolecules (2018) 8(1):4. doi: 10.3390/biom8010004 PMC5871973 29351202

[58] Zhang Q-Y Yan Z-B Meng Y-M Hong X-Y Shao G Ma J-J. Antimicrobial Peptides: Mechanism of Action, Activity and Clinical Potential. Mil Med Res (2021) 8(1):1–25. doi: 10.1186/s40779-021-00343-2 34496967 PMC8425997

[59] Graf M Wilson DN. Intracellular Antimicrobial Peptides Targeting the Protein Synthesis Machinery. Antimicrob Pept (2019) 1117:73–89. doi: 10.1007/978-981-13-3588-4_6 30980354

[60] Yasir M Willcox MDP Dutta D. Action of Antimicrobial Peptides Against Bacterial Biofilms. Materials (2018) 11(12):2468. doi: 10.3390/ma11122468 PMC6317029 30563067

[61] Hanahan D Weinberg RA. Hallmarks of Cancer: The Next Generation. cell (2011) 144(5):646–74. doi: 10.1016/j.cell.2011.02.013 21376230

[62] Zalba S Ten Hagen TL. Cell Membrane Modulation as Adjuvant in Cancer Therapy. Cancer Treat Rev (2017) 52:48–57. doi: 10.1016/j.ctrv.2016.10.008 27889637 PMC5195909

[63] Ran S Downes A Thorpe PE. Increased Exposure of Anionic Phospholipids on the Surface of Tumor Blood Vessels. Cancer Res (2002) 62(21):6132–40. 12414638

[64] Leite NB Aufderhorst-Roberts A Palma MS Connell SD Neto JR Beales PA. PE and PS Lipids Synergistically Enhance Membrane Poration by a Peptide With Anticancer Properties. Biophys J (2015) 109(5):936–47. doi: 10.1016/j.bpj.2015.07.033 PMC4564682 26331251

[65] Teixeira V Feio MJ Bastos M. Role of Lipids in the Interaction of Antimicrobial Peptides With Membranes. Prog Lipid Res (2012) 51(2):149–77. doi: 10.1016/j.plipres.2011.12.005 22245454

[66] Teleanu RI Chircov C Grumezescu AM Teleanu DM. Tumor Angiogenesis and Anti-Angiogenic Strategies for Cancer Treatment. J Clin Med (2020) 9(1):84. doi: 10.3390/jcm9010084 PMC7020037 31905724

[67] Semenza GL. Regulation of Cancer Cell Metabolism by Hypoxia-Inducible Factor 1. Semin Cancer Biol (2009) 19(1):12–6. doi: 10.1016/j.semcancer.2008.11.009 19114105

[68] Jafari A Babajani A Abdollahpour-Alitappeh M Ahmadi N Rezaei-Tavirani M. Exosomes and Cancer: From Molecular Mechanisms to Clinical Applications. Med Oncol (2021) 38(4):1–17. doi: 10.1007/s12032-021-01491-0 33743101

[69] Zhao X Gao S Ren H Sun W Zhang H Sun J. Hypoxia-Inducible Factor-1 Promotes Pancreatic Ductal Adenocarcinoma Invasion and Metastasis by Activating Transcription of the Actin-Bundling Protein Fascin. Cancer Res (2014) 74(9):2455–64. doi: 10.1158/0008-5472.CAN-13-3009 24599125

[70] Azab AK Hu J Quang P Azab F Pitsillides C Awwad R. Hypoxia Promotes Dissemination of Multiple Myeloma Through Acquisition of Epithelial to Mesenchymal Transition-Like Features. Blood J Am Soc Hematol (2012) 119(24):5782–94. doi: 10.1182/blood-2011-09-380410 PMC3382938 22394600

[71] Jiang X Wang J Deng X Xiong F Zhang S Gong Z. The Role of Microenvironment in Tumor Angiogenesis. J Exp Clin Cancer Res (2020) 39(1):204. doi: 10.1186/s13046-020-01709-5 32993787 PMC7526376

[72] Jang J-H Kim D-H Surh Y-J. Dynamic Roles of Inflammasomes in Inflammatory Tumor Microenvironment. NPJ Precis Oncol (2021) 5(1):18. doi: 10.1038/s41698-021-00154-7 33686176 PMC7940484

[73] Vasan N Baselga J Hyman DM. A View on Drug Resistance in Cancer. Nature (2019) 575(7782):299–309. doi: 10.1038/s41586-019-1730-1 31723286 PMC8008476

[74] Phi LTH Sari IN Yang YG Lee SH Jun N Kim KS. Cancer Stem Cells (CSCs) in Drug Resistance and Their Therapeutic Implications in Cancer Treatment. Stem Cells Int (2018) 2018:5416923. doi: 10.1155/2018/5416923 29681949 PMC5850899

[75] Pearce A Haas M Viney R Pearson S-A Haywood P Brown C. Incidence and Severity of Self-Reported Chemotherapy Side Effects in Routine Care: A Prospective Cohort Study. PloS One (2017) 12(10):e0184360. doi: 10.1371/journal.pone.0184360 29016607 PMC5634543

[76] Hoskin DW Ramamoorthy A. Studies on Anticancer Activities of Antimicrobial Peptides. Biochim Biophys Acta (BBA)-Biomembr (2008) 1778(2):357–75. doi: 10.1016/j.bbamem.2007.11.008 PMC2238813 18078805

[77] Chiangjong W Chutipongtanate S Hongeng S. Anticancer Peptide: Physicochemical Property, Functional Aspect and Trend in Clinical Application. Int J Oncol (2020) 57(3):678–96. doi: 10.3892/ijo.2020.5099 PMC7384845 32705178

[78] Balasubramanian K Schroit AJ. Aminophospholipid Asymmetry: A Matter of Life and Death. Annu Rev Physiol (2003) 65(1):701–34. doi: 10.1146/annurev.physiol.65.092101.142459 12471163

[79] Ran S Thorpe PE. Phosphatidylserine is a Marker of Tumor Vasculature and a Potential Target for Cancer Imaging and Therapy. Int J Radiat Oncol Biol Phys (2002) 54(5):1479–84. doi: 10.1016/S0360-3016(02)03928-7 12459374

[80] Kufe DW. Mucins in Cancer: Function, Prognosis and Therapy. Nat Rev Cancer (2009) 9(12):874–85. doi: 10.1038/nrc2761 PMC2951677 19935676

[81] Raman K Kuberan B. Chemical Tumor Biology of Heparan Sulfate Proteoglycans. Curr Chem Biol (2010) 4(1):20–31. doi: 10.2174/187231310790226206 20596243 PMC2892923

[82] Guterstam P Madani F Hirose H Takeuchi T Futaki S Andaloussi SE. Elucidating Cell-Penetrating Peptide Mechanisms of Action for Membrane Interaction, Cellular Uptake, and Translocation Utilizing the Hydrophobic Counter-Anion Pyrenebutyrate. Biochim Biophys Acta (BBA)-Biomembr (2009) 1788(12):2509–17. doi: 10.1016/j.bbamem.2009.09.014 19796627

[83] Pushpanathan M Rajendhran J Jayashree S Sundarakrishnan B Jayachandran S Gunasekaran P. Direct Cell Penetration of the Antifungal Peptide, MMGP1, in Candida Albicans. J Pept Sci (2012) 18(11):657–60. doi: 10.1002/psc.2445 23080316

[84] Park CB Yi K-S Matsuzaki K Kim MS Kim SC. Structure–activity Analysis of Buforin II, a Histone H2A-Derived Antimicrobial Peptide: The Proline Hinge is Responsible for the Cell-Penetrating Ability of Buforin II. Proc Natl Acad Sci (2000) 97(15):8245–50. doi: 10.1073/pnas.150518097 PMC26932 10890923

[85] Harris F Dennison SR Singh J Phoenix DA. On the Selectivity and Efficacy of Defense Peptides With Respect to Cancer Cells. Med Res Rev (2013) 33(1):190–234. doi: 10.1002/med.20252 21922503

[86] Schweizer F. Cationic Amphiphilic Peptides With Cancer-Selective Toxicity. Eur J Pharmacol (2009) 625(1-3):190–4. doi: 10.1016/j.ejphar.2009.08.043 19835863

[87] Agrawal P Bhagat D Mahalwal M Sharma N Raghava GP. AntiCP 2.0: An Updated Model for Predicting Anticancer Peptides. Briefings Bioinf (2021) 22(3):bbaa153. doi: 10.1093/bib/bbaa153 32770192

[88] Patel VG Oh WK Galsky MD. Treatment of Muscle-Invasive and Advanced Bladder Cancer in 2020. CA Cancer J Clin (2020) 70(5):404–23. doi: 10.3322/caac.21631 32767764

[89] Richters A Aben KK Kiemeney LA. The Global Burden of Urinary Bladder Cancer: An Update. World J Urol (2020) 38(8):1895–904. doi: 10.1007/s00345-019-02984-4 PMC7363726 31676912

[90] Peyton CC Tang D Reich RR Azizi M Chipollini J Pow-Sang JM. Downstaging and Survival Outcomes Associated With Neoadjuvant Chemotherapy Regimens Among Patients Treated With Cystectomy for Muscle-Invasive Bladder Cancer. JAMA Oncol (2018) 4(11):1535–42. doi: 10.1001/jamaoncol.2018.3542 PMC6248089 30178038

[91] Suttmann H Retz M Paulsen F Harder J Zwergel U Kamradt J. Antimicrobial Peptides of the Cecropin-Family Show Potent Antitumor Activity Against Bladder Cancer Cells. BMC Urol (2008) 8(1):1–7. doi: 10.1186/1471-2490-8-5 18315881 PMC2276511

[92] Wang K-R Zhang B-Z Zhang W Yan J-X Li J Wang R. Antitumor Effects, Cell Selectivity and Structure-Activity Relationship of a Novel Antimicrobial Peptide Polybia-MPI. Peptides (2008) 29(6):963–8. doi: 10.1016/j.peptides.2008.01.015 18328599

[93] Li G Lei Q Wang F Deng D Wang S Tian L. Fluorinated Polymer Mediated Transmucosal Peptide Delivery for Intravesical Instillation Therapy of Bladder Cancer. Small (2019) 15(25):1900936. doi: 10.1002/smll.201900936 31074941

[94] Huang H-N Rajanbabu V Pan C-Y Chan Y-L Wu C-J Chen J-Y. A Cancer Vaccine Based on the Marine Antimicrobial Peptide Pardaxin (GE33) for Control of Bladder-Associated Tumors. Biomaterials (2013) 34(38):10151–9. doi: 10.1016/j.biomaterials.2013.09.041 24075482

[95] Mintz J Vedenko A Rosete O Shah K Goldstein G Hare JM. Current Advances of Nitric Oxide in Cancer and Anticancer Therapeutics. Vaccines (2021) 9(2):94. doi: 10.3390/vaccines9020094 33513777 PMC7912608

[96] Slamon DJ Leyland-Jones B Shak S Fuchs H Paton V Bajamonde A. Use of Chemotherapy Plus a Monoclonal Antibody Against HER2 for Metastatic Breast Cancer That Overexpresses HER2. New Engl J Med (2001) 344(11):783–92. doi: 10.1056/NEJM200103153441101 11248153

[97] Bao T Rudek MA. The Clinical Pharmacology of Anastrozole. Eur Oncol Haematol (2011) 7(2):106–8. doi: 10.17925/EOH.2011.07.02.106

[98] Vargas Casanova Y Rodriguez Guerra JA Umana Perez YA Leal Castro AL Almanzar Reina G Garcia Castaneda JE. Antibacterial Synthetic Peptides Derived From Bovine Lactoferricin Exhibit Cytotoxic Effect Against MDA-MB-468 and MDA-MB-231 Breast Cancer Cell Lines. Molecules (2017) 22(10):1641. doi: 10.3390/molecules22101641 PMC6151437 28961215

[99] E-Kobon T Thongararm P Roytrakul S Meesuk L Chumnanpuen P. Prediction of Anticancer Peptides Against MCF-7 Breast Cancer Cells From the Peptidomes of Achatina Fulica Mucus Fractions. Comput Struct Biotechnol J (2016) 14:49–57. doi: 10.1016/j.csbj.2015.11.005 26862373 PMC4706611

[100] Hsiao Y-C Wang K-S Tsai S-H Chao W-T Lung F-DT. Anticancer Activities of an Antimicrobial Peptide Derivative of Ixosin-B Amide. Bioorg Med Chem Lett (2013) 23(20):5744–7. doi: 10.1016/j.bmcl.2013.07.063 23993331

[101] Wu Y-S Liao Z-J Wang K-S Lung F-DT. Structure–activity Relationship of Potent Antimicrobial Peptide Analogs of Ixosin-B Amide. Bioorg Med Chem Lett (2013) 23(10):2929–32. doi: 10.1016/j.bmcl.2013.03.053 23570790

[102] Smetanin M Sek S Maran F Lipkowski J. Molecular Resolution Visualization of a Pore Formed by Trichogin, an Antimicrobial Peptide, in a Phospholipid Matrix. Biochim Biophys Acta (BBA)-Biomembr (2014) 1838(12):3130–6. doi: 10.1016/j.bbamem.2014.08.006 25157669

[103] Guzmán-Rodríguez JJ López-Gómez R Salgado-Garciglia R Ochoa-Zarzosa A López-Meza JE. The Defensin From Avocado (Persea Americana Var. Drymifolia) PaDef Induces Apoptosis in the Human Breast Cancer Cell Line MCF-7. Biomed Pharmacother (2016) 82:620–7. doi: 10.1016/j.biopha.2016.05.048 27470405

[104] Ting C-H Chen Y-C Wu C-J Chen J-Y. Targeting FOSB With a Cationic Antimicrobial Peptide, TP4, for Treatment of Triple-Negative Breast Cancer. Oncotarget (2016) 7(26):40329. doi: 10.18632/oncotarget.9612 27248170 PMC5130011

[105] Ameyar M Wisniewska M Weitzman JB. A Role for AP-1 in Apoptosis: The Case for and Against. Biochimie (2003) 85(8):747–52. doi: 10.1016/j.biochi.2003.09.006 14585541

[106] Bossy-Wetzel E Bakiri L Yaniv M. Induction of Apoptosis by the Transcription Factor C-Jun. EMBO J (1997) 16(7):1695–709. doi: 10.1093/emboj/16.7.1695 PMC1169773 9130714

[107] Park J-A Na H-H Jin H-O Kim K-C. Increased Expression of FosB Through Reactive Oxygen Species Accumulation Functions as Pro-Apoptotic Protein in Piperlongumine Treated MCF7 Breast Cancer Cells. Mol Cells (2019) 42(12):884. doi: 10.14348/molcells.2019.0088 31735020 PMC6939652

[108] Wang C Zhou Y Li S Li H Tian L Wang H. Anticancer Mechanisms of Temporin-1cea, an Amphipathic α-Helical Antimicrobial Peptide, in Bcap-37 Human Breast Cancer Cells. Life Sci (2013) 92(20-21):1004–14. doi: 10.1016/j.lfs.2013.03.016 23583573

[109] Hilchie AL Doucette CD Pinto DM Patrzykat A Douglas S Hoskin DW. Pleurocidin-Family Cationic Antimicrobial Peptides are Cytolytic for Breast Carcinoma Cells and Prevent Growth of Tumor Xenografts. Breast Cancer Res (2011) 13(5):1–16. doi: 10.1186/bcr3043 PMC3262215 22023734

[110] Avand A Akbari V Shafizadegan S. *In Vitro* Cytotoxic Activity of a Lactococcus Lactis Antimicrobial Peptide Against Breast Cancer Cells. Iran J Biotechnol (2018) 16(3):231–20. doi: 10.21859/ijb.1867 PMC6697826 31457026

[111] Arroyo JMG López MLD. Psychological Problems Derived From Mastectomy: A Qualitative Study. Int J Surg Oncol (2011) 2011:132461. doi: 10.1155/2011/132461 22312492 PMC3265278

[112] Siegel RL Miller KD Goding Sauer A Fedewa SA Butterly LF Anderson JC. Colorectal Cancer Statistics, 2020. CA: Cancer J Clin (2020) 70(3):145–64. doi: 10.3322/caac.21601 32133645

[113] Mármol I Sánchez-de-Diego C Pradilla Dieste A Cerrada E Rodriguez Yoldi MJ. Colorectal Carcinoma: A General Overview and Future Perspectives in Colorectal Cancer. Int J Mol Sci (2017) 18(1):197. doi: 10.3390/ijms18010197 PMC5297828 28106826

[114] Arpornsuwan T Sriwai W Jaresitthikunchai J Phaonakrop N Sritanaudomchai H Roytrakul S. Anticancer Activities of Antimicrobial BmKn2 Peptides Against Oral and Colon Cancer Cells. Int J Pept Res Ther (2014) 20(4):501–9. doi: 10.1007/s10989-014-9417-9

[115] Varas MA Muñoz-Montecinos C Kallens V Simon V Allende ML Marcoleta AE. Exploiting Zebrafish Xenografts for Testing the *In Vivo* Antitumorigenic Activity of Microcin E492 Against Human Colorectal Cancer Cells. Front Microbiol (2020) 11:405. doi: 10.3389/fmicb.2020.00405 32265865 PMC7096547

[116] Norouzi Z Salimi A Halabian R Fahimi H. Nisin, a Potent Bacteriocin and Anti-Bacterial Peptide, Attenuates Expression of Metastatic Genes in Colorectal Cancer Cell Lines. Microb Pathog (2018) 123:183–9. doi: 10.1016/j.micpath.2018.07.006 30017942

[117] Kuroda K Fukuda T Isogai H Okumura K Krstic-Demonacos M Isogai E. Antimicrobial Peptide FF/CAP18 Induces Apoptotic Cell Death in HCT116 Colon Cancer Cells *via* Changes in the Metabolic Profile. Int J Oncol (2015) 46(4):1516–26. doi: 10.3892/ijo.2015.2887 PMC4356497 25672949

[118] Lewies A Wentzel JF Miller HC Du Plessis LH. The Antimicrobial Peptide Nisin Z Induces Selective Toxicity and Apoptotic Cell Death in Cultured Melanoma Cells. Biochimie (2018) 144:28–40. doi: 10.1016/j.biochi.2017.10.009 29054798

[119] Zamaraeva M Sabirov R Maeno E Ando-Akatsuka Y Bessonova S Okada Y. Cells Die With Increased Cytosolic ATP During Apoptosis: A Bioluminescence Study With Intracellular Luciferase. Cell Death Differ (2005) 12(11):1390–7. doi: 10.1038/sj.cdd.4401661 15905877

[120] Daghero H Fernández Massó JR Astrada S Guerra Vallespí M Bollati-Fogolín M. The Anticancer Peptide CIGB-552 Exerts Anti-Inflammatory and Anti-Angiogenic Effects Through COMMD1. Molecules (2021) 26(1):152. doi: 10.3390/molecules26010152 PMC7795859 33396282

[121] Jiang R Lönnerdal B. Bovine Lactoferrin and Lactoferricin Exert Antitumor Activities on Human Colorectal Cancer Cells (HT-29) by Activating Various Signaling Pathways. Biochem Cell Biol (2017) 95(1):99–109. doi: 10.1139/bcb-2016-0094 28169560

[122] Kuroda K Fukuda T Yoneyama H Katayama M Isogai H Okumura K. Anti-Proliferative Effect of an Analogue of the LL-37 Peptide in the Colon Cancer Derived Cell Line HCT116 P53+/+ and P53. Oncol Rep (2012) 28(3):829–34. doi: 10.3892/or.2012.1876 22736062

[123] Wu K-L Tsai Y-M Lien C-T Kuo P-L Hung J-Y. The Roles of MicroRNA in Lung Cancer. Int J Mol Sci (2019) 20(7):1611. doi: 10.3390/ijms20071611 PMC6480472 30935143

[124] Kuroda K Fukuda T Krstic-Demonacos M Demonacos C Okumura K Isogai H. miR-663a Regulates Growth of Colon Cancer Cells, After Administration of Antimicrobial Peptides, by Targeting CXCR4-P21 Pathway. BMC Cancer (2017) 17(1):1–10. doi: 10.1186/s12885-016-3003-9 28061765 PMC5219750

[125] Maijaroen S Jangpromma N Daduang J Klaynongsruang S. KT2 and RT2 Modified Antimicrobial Peptides Derived From Crocodylus Siamensis Leucrocin I Show Activity Against Human Colon Cancer HCT-116 Cells. Environ Toxicol Pharmacol (2018) 62:164–76. doi: 10.1016/j.etap.2018.07.007 30031283

[126] Fan R Tong A Li X Gao X Mei L Zhou L. Enhanced Antitumor Effects by Docetaxel/LL37-Loaded Thermosensitive Hydrogel Nanoparticles in Peritoneal Carcinomatosis of Colorectal Cancer. Int J Nanomed (2015) 10:7291. doi: 10.2147/IJN.S89066 PMC4672756 26664119

[127] Chauhan S Dhawan DK Saini A Preet S. Antimicrobial Peptides Against Colorectal Cancer-A Focused Review. Pharmacol Res (2021) 105529:1–29. doi: 10.1016/j.phrs.2021.105529 33675962

[128] Bleeker FE Molenaar RJ Leenstra S. Recent Advances in the Molecular Understanding of Glioblastoma. J Neuro-Oncol (2012) 108(1):11–27. doi: 10.1007/s11060-011-0793-0 PMC3337398 22270850

[129] Tewarie IA Senders JT Kremer S Devi S Gormley WB Arnaout O. Survival Prediction of Glioblastoma Patients-Are We There Yet? A Systematic Review of Prognostic Modeling for Glioblastoma and Its Clinical Potential. Neurosurg Rev (2021) 44(4):2047–57. doi: 10.1007/s10143-020-01430-z PMC8338817 33156423

[130] Guo X Ma C Du Q Wei R Wang L Zhou M. Two Peptides, TsAP-1 and TsAP-2, From the Venom of the Brazilian Yellow Scorpion, Tityus Serrulatus: Evaluation of Their Antimicrobial and Anticancer Activities. Biochimie (2013) 95(9):1784–94. doi: 10.1016/j.biochi.2013.06.003 23770440

[131] Shi D Hou X Wang L Gao Y Wu D Xi X. Two Novel Dermaseptin-Like Antimicrobial Peptides With Anticancer Activities From the Skin Secretion of Pachymedusa Dacnicolor. Toxins (2016) 8(5):144. doi: 10.3390/toxins8050144 PMC4885059 27187467

[132] Su B-C Wu T-H Hsu C-H Chen J-Y. Distribution of Positively Charged Amino Acid Residues in Antimicrobial Peptide Epinecidin-1 Is Crucial for *In Vitro* Glioblastoma Cytotoxicity and Its Underlying Mechanisms. Chemico-biol Interact (2020) 315:108904. doi: 10.1016/j.cbi.2019.108904 31758921

[133] Chen X Pan Y Yan M Bao G Sun X. Identification of Potential Crucial Genes and Molecular Mechanisms in Glioblastoma Multiforme by Bioinformatics Analysis. Mol Med Rep (2020) 22(2):859–69. doi: 10.3892/mmr.2020.11160 PMC7339479 32467990

[134] Cuddapah VA Robel S Watkins S Sontheimer H. A Neurocentric Perspective on Glioma Invasion. Nat Rev Neurosci (2014) 15(7):455–65. doi: 10.1038/nrn3765 PMC5304245 24946761

[135] Chen YF Shih PC Kuo HM Yang SN Lin YY Chen WF. TP3, an Antimicrobial Peptide, Inhibits Infiltration and Motility of Glioblastoma Cells *via* Modulating the Tumor Microenvironment. Cancer Med (2020) 9(11):3918–31. doi: 10.1002/cam4.3005 PMC7286473 32266797

[136] Su B-C Pan C-Y Chen J-Y. Antimicrobial Peptide TP4 Induces ROS-Mediated Necrosis by Triggering Mitochondrial Dysfunction in Wild-Type and Mutant P53 Glioblastoma Cells. Cancers (2019) 11(2):171. doi: 10.3390/cancers11020171 PMC6406555 30717309

[137] Su B-C Mo F-E. CCN1 Enables Fas Ligand-Induced Apoptosis in Cardiomyoblast H9c2 Cells by Disrupting Caspase Inhibitor XIAP. Cell Signal (2014) 26(6):1326–34. doi: 10.1016/j.cellsig.2014.02.019 24631528

[138] Katoh I Sato S Fukunishi N Yoshida H Imai T Kurata S-I. Apaf-1-Deficient Fog Mouse Cell Apoptosis Involves Hypo-Polarization of the Mitochondrial Inner Membrane, ATP Depletion and Citrate Accumulation. Cell Res (2008) 18(12):1210–9. doi: 10.1038/cr.2008.87 18663378

[139] Abdel-Salam MA Carvalho-Tavares J Gomes KS Teixeira-Carvalho A Kitten GT Nyffeler J. The Synthetic Peptide LyeTxI-B Derived From Lycosa Erythrognatha Spider Venom Is Cytotoxic to U-87 MG Glioblastoma Cells. Amino Acids (2019) 51(3):433–49. doi: 10.1007/s00726-018-2678-4 30449002

[140] Liu Y Cao X. Intratumoral Dendritic Cells in the Anti-Tumor Immune Response. Cell Mol Immunol (2015) 12(4):387–90. doi: 10.1038/cmi.2014.130 PMC4496534 25597333

[141] Hou J Zhang Q Liu Z Wang S Li D Liu C. Cyclophilin A as a Potential Genetic Adjuvant to Improve HIV-1 Gag DNA Vaccine Immunogenicity by Eliciting Broad and Long-Term Gag-Specific Cellular Immunity in Mice. Vaccin Immunother (2016) 12(2):545–53. doi: 10.1080/21645515.2015.1082692 PMC5049736 26305669

[142] Jana A Narula P Chugh A Kulshreshtha R. Efficient Delivery of anti-miR-210 Using Tachyplesin, a Cell Penetrating Peptide, for Glioblastoma Treatment. Int J Pharm (2019) 572:118789. doi: 10.1016/j.ijpharm.2019.118789 31726199

[143] Agrawal R Garg A Benny Malgulwar P Sharma V Sarkar C Kulshreshtha R. P53 and miR-210 Regulated NeuroD2, a Neuronal Basic Helix-Loop-Helix Transcription Factor, Is Downregulated in Glioblastoma Patients and Functions as a Tumor Suppressor Under Hypoxic Microenvironment. Int J Cancer (2018) 142(9):1817–28. doi: 10.1002/ijc.31209 29226333

[144] Su B-C Chen J-Y. Pharmacological Inhibition of P38 Potentiates Antimicrobial Peptide TP4-Induced Cell Death in Glioblastoma Cells. Mol Cell Biochem (2020) 464(1):1–9. doi: 10.1007/s11010-019-03643-3 31673920

[145] Ma L Liu J Zhang X Qi J Yu W Gu Y. P38 MAPK-Dependent Nrf2 Induction Enhances the Resistance of Glioma Cells Against TMZ. Med Oncol (2015) 32(3):69. doi: 10.1007/s12032-015-0517-y 25691294

[146] Yoshino Y Aoyagi M Tamaki M Duan L Morimoto T Ohno K. Activation of P38 MAPK and/or JNK Contributes to Increased Levels of VEGF Secretion in Human Malignant Glioma Cells. Int J Oncol (2006) 29(4):981–7. doi: 10.3892/ijo.29.4.981 16964394

[147] Munoz L Yeung YT Grewal T. Oncogenic Ras Modulates P38 MAPK-Mediated Inflammatory Cytokine Production in Glioblastoma Cells. Cancer Biol Ther (2016) 17(4):355–63. doi: 10.1080/15384047.2016.1139249 PMC4910925 26794430

[148] Sangpairoj K Vivithanaporn P Apisawetakan S Chongthammakun S Sobhon P Chaithirayanon K. RUNX1 Regulates Migration, Invasion, and Angiogenesis *via* P38 MAPK Pathway in Human Glioblastoma. Cell Mol Neurobiol (2017) 37(7):1243–55. doi: 10.1007/s10571-016-0456-y PMC11482080 28012022

[149] Bray F Ferlay J Soerjomataram I Siegel RL Torre LA Jemal A. Global Cancer Statistics 2018: GLOBOCAN Estimates of Incidence and Mortality Worldwide for 36 Cancers in 185 Countries. CA: Cancer J Clin (2018) 68(6):394–424. doi: 10.3322/caac.21492 30207593

[150] Lee W-H Loo C-Y Ghadiri M Leong C-R Young PM Traini D. The Potential to Treat Lung Cancer *via* Inhalation of Repurposed Drugs. Adv Drug Deliv Rev (2018) 133:107–30. doi: 10.1016/j.addr.2018.08.012 30189271

[151] Kaushik AC Mehmood A Wei D-Q Dai X. Systems Biology Integration and Screening of Reliable Prognostic Markers to Create Synergies in the Control of Lung Cancer Patients. Front Mol Biosci (2020) 7(47). doi: 10.3389/fmolb.2020.00047 PMC7154114 32318583

[152] Swithenbank L Cox P Harris LG Dudley E Sinclair K Lewis P. Temporin A and Bombinin H2 Antimicrobial Peptides Exhibit Selective Cytotoxicity to Lung Cancer Cells. Scientifica (2020) 2020:1–10. doi: 10.1155/2020/3526286 PMC7341413 32676212

[153] Wu X Pan J Wu Y Xi X Ma C Wang L. PSN-PC: A Novel Antimicrobial and Anti-Biofilm Peptide From the Skin Secretion of Phyllomedusa-Camba With Cytotoxicity on Human Lung Cancer Cell. Molecules (2017) 22(11):1896. doi: 10.3390/molecules22111896 PMC6150266 29112170

[154] Liu J Wu Q Li L Xi X Wu D Zhou M. Discovery of Phylloseptins That Defense Against Gram-Positive Bacteria and Inhibit the Proliferation of the non-Small Cell Lung Cancer Cell Line, From the Skin Secretions of Phyllomedusa Frogs. Molecules (2017) 22(9):1428. doi: 10.3390/molecules22091428 PMC6151776 28850103

[155] Huang C-Y Huang H-Y Forrest MD Pan Y-R Wu W-J Chen H-M. Inhibition Effect of a Custom Peptide on Lung Tumors. PloS One (2014) 9(10):e109174. doi: 10.1371/journal.pone.0109174 25310698 PMC4195615

[156] Ting C-H Liu Y-C Lyu P-C Chen J-Y. Nile Tilapia Derived Antimicrobial Peptide TP4 Exerts Antineoplastic Activity Through Microtubule Disruption. Mar Drugs (2018) 16(12):462. doi: 10.3390/md16120462 PMC6315541 30469546

[157] Ting C-H Lee K-Y Wu S-M Feng P-H Chan Y-F Chen Y-C. FOSB–PCDHB13 Axis Disrupts the Microtubule Network in non-Small Cell Lung Cancer. Cancers (2019) 11(1):107. doi: 10.3390/cancers11010107 PMC6357195 30658436

[158] Wang S Tu J Zhou C Li J Huang L Tao L. The Effect of Lfcin-B on Non-Small Cell Lung Cancer H460 Cells Is Mediated by Inhibiting VEGF Expression and Inducing Apoptosis. Arch Pharmacal Res (2015) 38(2):261–71. doi: 10.1007/s12272-014-0373-x 24691828

[159] Banerjee S Parasramka MA Paruthy SB. Garcinol: Preclinical Perspective Underpinning Chemo- and Radiosensitization of Cancer. Role Nutraceuticals Chemoresistance to Cancer: Elsevier; (2018) p:297–324. doi: 10.1016/B978-0-12-812373-7.00015-2

[160] Keykhaei M Masinaei M Mohammadi E Azadnajafabad S Rezaei N Saeedi Moghaddam S. A Global, Regional, and National Survey on Burden and Quality of Care Index (QCI) of Hematologic Malignancies; Global Burden of Disease Systematic Analysis 1990-2017. Exp Hematol Oncol (2021) 10(1):11. doi: 10.1186/s40164-021-00198-2 33557940 PMC7869509

[161] Elrayess RA Mohallal ME El-Shahat YM Ebaid HM Miller K Strong PN. Cytotoxic Effects of Smp24 and Smp43 Scorpion Venom Antimicrobial Peptides on Tumour and non-Tumour Cell Lines. Int J Pept Res Ther (2020) 26(3):1409–15. doi: 10.1007/s10989-019-09932-1

[162] Hilchie AL Conrad DM Power Coombs MR Zemlak T Doucette CD Liwski RS. Pleurocidin-Family Cationic Antimicrobial Peptides Mediate Lysis of Multiple Myeloma Cells and Impair the Growth of Multiple Myeloma Xenografts. Leukemia lymphoma (2013) 54(10):2255–62. doi: 10.3109/10428194.2013.770847 23350892

[163] Mader JS Salsman J Conrad DM Hoskin DW. Bovine Lactoferricin Selectively Induces Apoptosis in Human Leukemia and Carcinoma Cell Lines. Mol Cancer Ther (2005) 4(4):612–24. doi: 10.1158/1535-7163.MCT-04-0077 15827335

[164] Sevrioukova IF. Apoptosis-Inducing Factor: Structure, Function, and Redox Regulation. Antioxid Redox Signal (2011) 14(12):2545–79. doi: 10.1089/ars.2010.3445 PMC3096518 20868295

[165] Mader JS Mookherjee N Hancock REW Bleackley RC. The Human Host Defense Peptide LL-37 Induces Apoptosis in a Calpain- and Apoptosis-Inducing Factor-Dependent Manner Involving Bax Activity. Mol Cancer Res (2009) 7(5):689–702. doi: 10.1158/1541-7786.MCR-08-0274 19435812

[166] Lv Y Shao G Zhang Q Wang X Meng Y Wang L. The Antimicrobial Peptide PFR Induces Necroptosis Mediated by ER Stress and Elevated Cytoplasmic Calcium and Mitochondrial ROS Levels: Cooperation With Ara-C to Act Against Acute Myeloid Leukemia. Signal transduction targeted Ther (2019) 4(1):1–3. doi: 10.1038/s41392-019-0073-6 PMC6799817 31637016

[167] Dennis M Burnett A Hills R Thomas I Ariti C Severinsen MT. A Randomised Evaluation of Low-Dose Cytosine Arabinoside (Ara-C) Plus Tosedostat Versus Low-Dose Ara-C in Older Patients With Acute Myeloid Leukaemia: Results of the LI-1 Trial. Br J Haematol (2021) bjh.17501:298–308. doi: 10.1111/bjh.17501 33961292

[168] Zhang J Gu Y Chen B. Mechanisms of Drug Resistance in Acute Myeloid Leukemia. OncoTargets Ther (2019) 12:1937. doi: 10.2147/OTT.S191621 PMC6417008 30881045

[169] Berge G Eliassen LT Camilio KA Bartnes K Sveinbjørnsson B Rekdal Ø. Therapeutic Vaccination Against a Murine Lymphoma by Intratumoral Injection of a Cationic Anticancer Peptide. Cancer Immunol Immunother (2010) 59(8):1285–94. doi: 10.1007/s00262-010-0857-6 PMC11030072 20422410

[170] van den Bijgaart RJ Schuurmans F Fütterer JJ Verheij M Cornelissen LA Adema GJ. Immune Modulation Plus Tumor Ablation: Adjuvants and Antibodies to Prime and Boost Anti-Tumor Immunity in Situ. Front Immunol (2021) 12:1156. doi: 10.3389/fimmu.2021.617365 PMC8079760 33936033

[171] Basu S Binder RJ Suto R Anderson KM Srivastava PK. Necrotic But Not Apoptotic Cell Death Releases Heat Shock Proteins, Which Deliver a Partial Maturation Signal to Dendritic Cells and Activate the NF-Kappa B Pathway. Int Immunol (2000) 12(11):1539–46. doi: 10.1093/intimm/12.11.1539 11058573

[172] Gordon YJ Romanowski EG McDermott AM. A Review of Antimicrobial Peptides and Their Therapeutic Potential as Anti-Infective Drugs. Curr Eye Res (2005) 30(7):505–15. doi: 10.1080/02713680590968637 PMC1497874 16020284

[173] Moore A. The Big and Small of Drug Discovery: Biotech Versus Pharma: Advantages and Drawbacks in Drug Development. EMBO Rep (2003) 4(2):114–7. doi: 10.1038/sj.embor.embor748 PMC1315844 12612596

[174] van der Velden WJ van Iersel TM Blijlevens NM Donnelly JP. Safety and Tolerability of the Antimicrobial Peptide Human Lactoferrin 1-11 (Hlf1-11). BMC Med (2009) 7(1):1–8. doi: 10.1186/1741-7015-7-44 19735580 PMC2746231

[175] Fjell CD Hiss JA Hancock RE Schneider G. Designing Antimicrobial Peptides: Form Follows Function. Nat Rev Drug Discov (2012) 11(1):37–51. doi: 10.1038/nrd3591 22173434

[176] Miyakoshi S Usuki K Matsumura I Ueda Y Iwasaki H Miyamoto T. Preliminary Results From a Phase 1/2 Study of DSP-7888, a Novel WT1 Peptide-Based Vaccine, in Patients With Myelodysplastic Syndrome (MDS). Blood (2016) 128(22):4335–. doi: 10.1182/blood.V128.22.4335.4335

[177] Starr CG Maderdrut JL He J Coy DH Wimley WC. Pituitary Adenylate Cyclase-Activating Polypeptide Is a Potent Broad-Spectrum Antimicrobial Peptide: Structure-Activity Relationships. Peptides (2018) 104:35–40. doi: 10.1016/j.peptides.2018.04.006 29654809 PMC5982112

[178] Mirski T Niemcewicz M Bartoszcze M Gryko R Michalski A. Utilisation of Peptides Against Microbial Infections - a Review. Ann Agric Environ Med (2017) 25(2):205–10. doi: 10.26444/aaem/74471 29936826

[179] Oddo A Hansen PR. Hemolytic Activity of Antimicrobial Peptides. Methods Mol Biol (2017) 1548:427–35. doi: 10.1007/978-1-4939-6737-7_31 28013523

[180] Staubitz P Peschel A Nieuwenhuizen WF Otto M Götz F Jung G. Structure–function Relationships in the Tryptophan-Rich, Antimicrobial Peptide Indolicidin. J Pept Sci: an Off Publ Eur Pept Soc (2001) 7(10):552–64. doi: 10.1002/psc.351 11695650

[181] Smirnova MP Kolodkin NI Kolobov AA Afonin VG Afonina IV Stefanenko LI. Indolicidin Analogs With Broad-Spectrum Antimicrobial Activity and Low Hemolytic Activity. Peptides (2020) 132:170356. doi: 10.1016/j.peptides.2020.170356 32593681

[182] Gaspar D Veiga AS Castanho MA. From Antimicrobial to Anticancer Peptides. A Review. Front Microbiol (2013) 4:294. doi: 10.3389/fmicb.2013.00294 24101917 PMC3787199

[183] Wang G. Post-Translational Modifications of Natural Antimicrobial Peptides and Strategies for Peptide Engineering. Curr Biotechnol (2012) 1(1):72–9. doi: 10.2174/2211550111201010072 PMC3914544 24511461

[184] Chen CH Lu TK. Development and Challenges of Antimicrobial Peptides for Therapeutic Applications. Antibiotics (2020) 9(1):24. doi: 10.3390/antibiotics9010024 PMC7168295 31941022

[185] Bakare OO Gokul A Wu R Niekerk L-A Klein A Keyster M. Biomedical Relevance of Novel Anticancer Peptides in the Sensitive Treatment of Cancer. Biomolecules (2021) 11(8):1120. doi: 10.3390/biom11081120 34439786 PMC8394746

[186] Huh AJ Kwon YJ. "Nanoantibiotics": A New Paradigm for Treating Infectious Diseases Using Nanomaterials in the Antibiotics Resistant Era. J Control Release (2011) 156(2):128–45. doi: 10.1016/j.jconrel.2011.07.002 21763369

[187] Malmsten M. Nanomaterials as Antimicrobial Agents. In: Bhushan B Luo D Schricker SR Sigmund W Zauscher S, editors. Handbook of Nanomaterials Properties. Berlin, Heidelberg: Springer Berlin Heidelberg (2014). p. 1053–75.

[188] Kenawy E-R Worley SD Broughton R. The Chemistry and Applications of Antimicrobial Polymers: A State-Of-the-Art Review. Biomacromolecules (2007) 8(5):1359–84. doi: 10.1021/bm061150q 17425365

[189] Jafari A Babajani A Rezaei-Tavirani M. Multiple Sclerosis Biomarker Discoveries by Proteomics and Metabolomics Approaches. Biomark Insights (2021) 16:11772719211013352. doi: 10.1177/11772719211013352 34017167 PMC8114757

[190] Fadnes B Uhlin-Hansen L Lindin I Rekdal Ø. Small Lytic Peptides Escape the Inhibitory Effect of Heparan Sulfate on the Surface of Cancer Cells. BMC Cancer (2011) 11(1):1–11. doi: 10.1186/1471-2407-11-116 21453492 PMC3080343

[191] Porto WF Silva ON Franco OL. Prediction and Rational Design of Antimicrobial Peptides. Protein struct (2012) 1:377–96. doi: 10.5772/38023

